# Single-cell multimodal analysis identifies common regulatory programs in synovial fibroblasts of rheumatoid arthritis patients and modeled TNF-driven arthritis

**DOI:** 10.1186/s13073-022-01081-3

**Published:** 2022-07-26

**Authors:** Marietta Armaka, Dimitris Konstantopoulos, Christos Tzaferis, Matthieu D. Lavigne, Maria Sakkou, Anastasios Liakos, Petros P. Sfikakis, Meletios A. Dimopoulos, Maria Fousteri, George Kollias

**Affiliations:** 1 Institute for Fundamental Biomedical Research, Biomedical Sciences Research Center “Alexander Fleming”, Vari, Greece; 2 Institute for Bioinnovation, Biomedical Sciences Research Center “Alexander Fleming”, Vari, Greece; 3 Center of New Biotechnologies & Precision Medicine, National and Kapodistrian University of Athens Medical School, Athens, Greece; 4 Institute of Molecular Biology & Biotechnology, FORTH, Heraklion, Crete Greece; 5 Department of Physiology, Medical School, National and Kapodistrian University of Athens, Athens, Greece; 6 First Department of Propaedeutic Internal Medicine, National and Kapodistrian University of Athens Medical School, Athens, Greece; 7 Joint Rheumatology Program, National and Kapodistrian University of Athens Medical School, Athens, Greece; 8 Department of Clinical Therapeutics, National and Kapodistrian University of Athens Medical School, Athens, Greece

## Abstract

**Background:**

Synovial fibroblasts (SFs) are specialized cells of the synovium that provide nutrients and lubricants for the proper function of diarthrodial joints. Recent evidence appreciates the contribution of SF heterogeneity in arthritic pathologies. However, the normal SF profiles and the molecular networks that govern the transition from homeostatic to arthritic SF heterogeneity remain poorly defined.

**Methods:**

We applied a combined analysis of single-cell (sc) transcriptomes and epigenomes (scRNA-seq and scATAC-seq) to SFs derived from naïve and *hTNFtg* mice (mice that overexpress human TNF, a murine model for rheumatoid arthritis), by employing the Seurat and ArchR packages. To identify the cellular differentiation lineages, we conducted velocity and trajectory analysis by combining state-of-the-art algorithms including scVelo, Slingshot, and PAGA. We integrated the transcriptomic and epigenomic data to infer gene regulatory networks using ArchR and custom-implemented algorithms. We performed a canonical correlation analysis-based integration of murine data with publicly available datasets from SFs of rheumatoid arthritis patients and sought to identify conserved gene regulatory networks by utilizing the SCENIC algorithm in the human arthritic scRNA-seq atlas.

**Results:**

By comparing SFs from healthy and *hTNFtg* mice, we revealed seven homeostatic and two disease-specific subsets of SFs. In healthy synovium, SFs function towards chondro- and osteogenesis, tissue repair, and immune surveillance. The development of arthritis leads to shrinkage of homeostatic SFs and favors the emergence of SF profiles marked by Dkk3 and Lrrc15 expression, functioning towards enhanced inflammatory responses and matrix catabolic processes. Lineage inference analysis indicated that specific Thy1+ SFs at the root of trajectories lead to the intermediate Thy1+/Dkk3+/Lrrc15+ SF states and culminate in a destructive and inflammatory Thy1− SF identity. We further uncovered epigenetically primed gene programs driving the expansion of these arthritic SFs, regulated by NFkB and new candidates, such as Runx1. Cross-species analysis of human/mouse arthritic SF data determined conserved regulatory and transcriptional networks.

**Conclusions:**

We revealed a dynamic SF landscape from health to arthritis providing a functional genomic blueprint to understand the joint pathophysiology and highlight the fibroblast-oriented therapeutic targets for combating chronic inflammatory and destructive arthritic disease.

**Supplementary Information:**

The online version contains supplementary material available at 10.1186/s13073-022-01081-3.

## Background

Chronic arthritides including rheumatoid arthritis (RA) are complex inflammatory disorders that primarily affect the diarthrodial joints causing high morbidity and mortality in human patients. Cells driving pathogenicity in the affected joints include an expanding mass of synovial fibroblasts (SFs) typically infiltrated by myeloid and lymphoid cells, which together contribute to the development of an invasive pannus that degrades the cartilage and promotes osteolysis [1, 2]. Early studies in transgenic mice have established a key role for TNF in driving the full pathogenic process [3, 4]. This was confirmed later in humans by the introduction of anti-TNF therapies that proved efficacious in neutralizing disease in a large percentage of RA patients [5]. Further genetic studies in murine arthritis models revealed that TNF signaling in SFs mediates persistent fibroblast activation and promotes pro-proliferative, immune-regulatory, and invasive characteristics. These functions are both necessary and sufficient to orchestrate the initiation and progression of the inflammatory and damaging pathology even in the absence of adaptive immune responses [6–8] qualifying SFs as key effector cells and crucial therapeutic targets in chronic arthritis.

The synovial membrane, a highly specialized, multifunctional connective tissue membrane comprising two anatomically distinct layers: lining SFs (LSFs) and the recently identified CX3CR1+ lining macrophages [9], forms a thin outer layer adjacent to the inmost structures consisting of sublining SFs (SLSFs), macrophages, adipose cells, nerves, and blood vessels [10]. SFs in the RA pro-inflammatory microenvironment acquire an aggressive phenotype, reminiscent of transformed migratory tumor-like cells [11]. They operate as immune-modulatory cells by secreting cytokines and chemokines and mediate cartilage destruction by over-expressing MMP1, MMP3, and MMP9 matrix metallo-proteases [12, 13] as well as the receptor activator of NF-κB ligand (RANKL/*Tnfsf11*), which causes excessive osteoclastogenesis leading to bone erosions [14, 15].

Histopathological and high-resolution transcriptomic analysis of RA-affected joints indicated that distinct fibroblasts subpopulations in the lining and the sublining synovial compartments are linked to specific disease features. Lining fibroblasts markers include podoplanin (PDPN) and Lubricin/Proteoglycan 4 (PRG4), whereas sublining SFs are characterized by high THY1 and PDPN expression. The RA SF subpopulations are characterized by differential expression of several markers such as CD34, VCAM1, FAP, and pro-inflammatory mediators, such as CXCL12, CCL2, and IL6 [16, 17]. More recent studies classified the fibroblasts found in the synovial lining zone as being predominantly responsible for driving articular damage, whereas fibroblasts located in the sublining layer express genes that function towards inflammation [18, 19]. Additional recent evidence revealed a dominant Notch-mediated interplay of perivascular SLSFs with endothelial cells, establishing a positional gradient of Thy1^high^ SLSFs towards Thy1^low^/Prg4^high^ LSFs and driving tissue inflammation [20]. Although these studies were instrumental in providing valuable insights into the classification of pathogenic SF subpopulations and their associated functions in the RA synovium, the homeostatic to pathological transitions of SFs and the molecular networks that drive them have remained unclear.

We chose to analyze at the single-cell (sc) level, the SFs derived from the *hTNFtg* mouse, a highly employed and a proof-of-concept model of RA predicting the success of anti-TNF therapies in RA and other inflammatory arthritides. The mice suffer from a TNF-dependent, inflammatory joint disease, affecting mainly the peripheral skeleton. The affected joints are characterized by progressive tissue degeneration and degradation, while the absence of the pathognomonic-for-spondyloarthritides anabolic events, such as the formation of osteophytes or the psoriatic arthritis-like feature of the nailbed attack, strongly suggests a RA-like phenotype. The disease is fully dependent on the TNF/TNFRI-dependent signaling on SFs, suggesting a prototypical system to explore the pathogenicity of fibroblasts. In this study, we aimed to uncouple and characterize the homeostatic and pathological functions of SFs. We undertook an integrative approach by combining sc transcriptomic (scRNA-seq) and chromatin accessibility data (scATAC-seq) to define the underlying molecular switches that determine the staging and progression of disease from healthy to early inflammatory and subsequent destructive synovial tissue. Our data reveal the early emergence and further expansion of distinct pathogenic SF subtypes characterized by specific differentially activated pathways and regulatory networks emanating from a progenitor state that appear repressed in the normal sublining synovium. Changes observed in SF stranscriptomes were highly correlated to chromatin accessibility alterations and cellular trajectory inference pinpointed to novel key transcription factors (TFs) and target genes driving the expansion of the pathogenic cell profiles at specific times and locations during *hTNFtg* disease progression. Lastly, alignment of our murine data with available human RA data uncovered a highly conserved core regulatory transcriptional program, validating our modeling approach and revealing a set of novel biomarkers specific to TNF-driven RA. Our results provide a solid translational potential to prioritize novel molecular and cellular targets specific for the pathogenic transitions of synovial fibroblasts in RA.

## Methods

### Mice

All mice were bred and maintained on CBAxC57Bl/6J genetic background in the animal facilities of the BSRC Alexander Fleming under specific pathogen-free conditions.

### Flow cytometry and fluorescence-activated cell sorting

Isolation of SFs was performed from both hind paws. The ankle joints were dissected, and the tissues were disaggregated by incubation for 30 min at 37 °C in an enzymatic digestion medium consisting of DMEM, 10%heat-inactivated FBS, collagenase (0.5 mg ml^−1^) from *Clostridium histolyticum* (Sigma, C5138) and 0.03 mg ml^−1^ DNase (Sigma, 9003-98-9). Upon washing the cells with PBS containing DNase, they were blocked in 1% BSA in PBS and Fc blocker (unlabelled anti-CD16/32, Biolegend 101302) for 10 min at 4 °C and stained with fluorophore-conjugated antibodies for 20 min at 4 °C (anti-Pdpn PE-Cy7, Biolegend 127411; anti-Thy1 A647, Biolegend 105318; anti-CD31 APC/Fire 750, Biolegend 102433; anti-CD45 APC-Cy7, Biolegend 103116; anti-Ter119 APC-A780, eBioscience 47-5921-80). After washing with PBS, cells were resuspended in FACS buffer (PBS, 1%BSA). Sorting of cells was performed with BD FACSAria III and the BD FACSDiva software, and dead cells were excluded by DAPI staining. Sorting gaiting for single-cell RNA-seq/ATAC-seq and bulk RNA-seq was different (Additional file 1: Fig. S1B). For sorted populations, purity and viability were determined by reanalysis for the target population based on cell surface markers immediately post-sorting. Purity was > 99% for each target population.

### Histopathology and immunofluorescence

Histological H&E staining was performed on the paraffin ankle joint sections as previously described [7]. For immunofluorescence, cryosections were probed with antibodies against Thy1 (Alexa Fluor 488 anti-mouse CD90.2 antibody, Biolegend 105315, or Alexa Fluor 647 anti-mouse CD90.2 antibody, Biolegend 105318, both clone, 30-H12), Clu (polyclonal rabbit anti-human CLU/Clusterin, LS-C331486, LSBio), Gdf10 (GDF10 polyclonal antibody, BS-5720R, Bioss antibodies), CD31 (APC rat anti-mouse CD31, 551262, BD Biosciences, clone MEC 13.3), Notch3 (anti-Notch3 antibody, ab23426, abcam), Comp (anti-COMP/cartilage oligomeric matrix protein antibody, ab231977, abcam), CD44 (FITC rat anti-mouse CD44, 553133, BD Biosciences, clone IM7), Dkk3 (anti-Dkk3 antibody, 10365-I-AP, ProteinTech), Runx1 (anti-Runx1/AML1 antibody, ab92336, abcam), and Prg4/Lubricin (anti-Lubricin/MSF antibody, ab28484, abcam). To visualize the stainings, the following secondary antibodies were applied: Alexa-Fluor 647-conjugated secondary antibodies (anti-rabbit, A21244, 1834794; anti-rat, A21247, 1719171; anti-mouse:, A21235, 1868116; and anti-hamster, A21451, 1572558, Invitrogen) and biotinylated secondary antibodies (anti-rat, BA-9400, and anti-rabbit, BA-1000). Images were acquired with a TCS SP8X White Light Laser confocal microscope (Leica) and with an Eclipse E800 (Nikon) microscope equipped with a Dxm1200F camera (Nikon). Imaging analysis was performed with the ImageJ/Fiji software (NIH).

### Droplet-based single-cell RNA sequencing

To avoid any sex bias effect in the analyses, mice of both genders were included to generate samples. Sorted live Pdpn^+^ CD45^−^ CD31^−^ Ter119^−^ synovial cells of the ankle joints of WT mice at the age of 4 weeks (*n* = 3) and *hTNFtg* mice at 2 different stages of the disease, early at 4 weeks (*n* = 3) and established at 8 weeks old mice (*n* = 3), were subjected to 10X Chromium Single Cell 3’ Solution v3. The platform was used to generate targeted 3000 single-cell gel bead emulsion per sample, loaded with an initial cell viability of 80%. The scRNA-seq libraries were prepared following the 10X Genomics user guide (Single Cell 3’ v3 reagent kits). After encapsulation, emulsions were transferred to a thermal cycler for RT. cDNA was purified and amplified with primers provided in the Single Cell 3’ reagents (10X Genomics). After purification with 0.6× SPRIselect beads (Beckman Coulter), cDNA quality and yield were evaluated using an Agilent Bionalyzer 2100. Using the provided enzyme fragmentation mix, the libraries were fragmented, end-repaired, and A-tailed. The products were cleaned using SPRIselect beads, and the adaptors provided in the kit were ligated. After cleaning the ligation products, libraries were amplified and indexed with unique sample index i7 through PCR amplification. Final libraries were double-sided cleaned, and their quality and size were evaluated using an Agilent Bioanalyzer 2100. Libraries were sequenced by pooling them in 1 lane on Illumina NextSeq 500 sequencer to a depth of 100 million reads each (one lane 75PE). The forward read included 28 bp for the 10X Barcode-UMI, followed by 8 bp i7 index (sample index) and 10 bp on the reverse read. Reads were converted to FASTQ format using mkfastq from cellranger v3 (10X genomics). Reads were then aligned to the mouse reference genome (mm10, Ensembl annotation release 91). The steps of read alignment, UMI counting, and aggregation of individual sample count matrices into a pooled single matrix were performed using the 10X Genomics Cell Ranger pipeline (v3). Since all samples were multiplexed in the same Chromium Chip, and sequenced in the same lane, factors of technical variability (batch effect) should not be present in the dataset.

### Computational analysis of single-cell RNA sequencing data

The DoubletFinder [21] and Seurat R packages [22, 23] were used for doublet detection and quality control of the cells. Cells containing less than 500 genes or more than 10% of reads mapped to the mitochondrial genome were excluded from further analysis. Downstream analysis of the data was performed using the functions of the Seurat package as described below. Normalization was performed using the NormalizeData function, with “LogNormalise” as the normalization method and 10,000 as the scaling factor. To identify the most variable genes, the FindVariableFeatures function was applied with mean.var.plot (mvp) as a selection method, and the rest of the parameters were set to default. Scaling of gene expression values was achieved by the scaleData function. Principal component analysis on scaled values of most highly variable genes, as identified in previous steps, was performed by the runPCA function. To find the optimal number of principal components to be used during the step of clustering and non-linear dimensionality reduction, SVD *k*-fold cross-validation was performed with dismo R library (https://cran.r-project.org/web/packages/dismo/index.html). For cell clustering, a graph-based clustering approach was followed, encompassing the construction of a *k*-nearest neighbor graph of the cells and the utilization of the Louvain community detection algorithm. The FindNeighbors and FindClusters functions were used to achieve that, the first with the parameter dims set to the range 1:25 and the second with the parameter resolution set to 0.6. tSNE, and UMAP non-linear dimensionality reduction methods were used for cell visualization in 2D through the functions runTSNE and runUmap using the optimal number of PCs = 25. For the identification of cluster marker genes, marker gene detection (Wilcoxon rank sum test, adjusted *p*-value based on Bonferroni correction using all features in the dataset, group 1 = cells belonging to the tested cluster, group 2 = rest of the cells) was performed with the FindAllMarkers function, excluding genes that exhibited less than 25% of expression in both cell groups or an absolute value of average log fold change less than 0.25. The same approach was followed in both pooled and individual sample analysis (in this analysis, only cells belonging to the analyzed sample were used). A gene set overrepresentation analysis was conducted using the R package clusterProfiler [24]. The lists of upregulated genes from each cluster (*p*-value < 0.01 and avgLFC ≥ 0.25), as identified in the previous step, were used as an input gene list. All the active genes of the dataset were considered as the background set of genes. “Biological processes” gene sets were used and obtained from the GO database. Enriched GO terms were considered those that showed an adjusted *p*-value < 0.05 and a gene count ≥ 3.

### Sub-clustering analysis

For the sub-clustering of the S4.a population, a new Seurat object was created containing only the cells originating from this cluster. The steps of scaling, highly variable gene identification, PCA analysis, and clustering were repeated leading to the detection of two sub-clusters (hS4a and iS4a). Sub-cluster labels of S4.a cells were transferred to the initial object containing all cells. Subsequently, D.E.A was conducted using the findAllMarkers function. Upregulated genes for the two sub-clusters were selected by applying the thresholds described in the previous paragraph. Functional enrichment analysis of GO biological processes was conducted with clusterProfiler [24].

### Trajectory analysis

RNA velocity analysis was conducted by using velocyto v.0.17 [25] and scVelo v.0.2.3 [26]. In particular, to count spliced and unspliced reads for each sample, the 10× velocyto pipeline was run in the filtered cellranger-generated BAM files, while for single-cell RNA velocity inference, the dynamical model of scVelo was applied. To predict the root and terminal states of the underlying Markov process, the respective scVelo functions were applied. The resulting root cells were used to infer the latent time ordering of the *hTNFtg* cells.

Following the results of RNA velocity analysis, the R package Slingshot [27] and python package PAGA [28] were utilized. To run Slingshot, UMAP coordinates were used, while clusters S2b and S5 were set as possible starting points. The produced minimum spanning tree supported the existence of a pathogenic branch comprising S2a, S2d, S4b, and S4a.

### Human scRNA-seq gene regulatory network (GRN) inference

To infer GRNs from the human integrated scRNA-seq data, the SCENIC [29] workflow was applied in the normalized expression matrix. Briefly, initially co-expressed genes were grouped using the arboreto python tool [30, 31]. Next, using CisTarget [32], all the inferred groups that included a transcription factor (TF) were considered as GRNs, while all genes with motif evidence of the respective TF in their regulatory space (hg38__refseq-r80__500bp_up_and_100bp_down_tss.mc9nr, hg38__refseq r80__10kb_up_and_down_tss.mc9nr.feather) were considered as valid TF targets. Finally, each formed regulon was scored in each cell, using AUCell [29].

### Integration of human datasets

For the integration of human data, three different publicly available datasets were used [16, 17, 19]: (1) Mizoguchi, F. et al. dataset: Single fibroblasts were isolated by flow cytometry (PTPRC (CD45)−, GYPA−, PECAM1 (CD31)−, and PDPN+) followed by sc library generation with the Smart-Seq2 protocol. The Illumina HiSeq 2500 platform was used for sequencing. RNA-seq expression data that support the findings of this study have been deposited in GEO with the primary accession code GSE109450 [16]. (2). Stephenson, W. et al. dataset: A 3D printed droplet microfluidic control instrument was used to separate single cells. Libraries were sequenced on the Illumina HiSeq 2500 platform. RNA sequencing data that support the findings of this study have been deposited in dbGaP with the accession code phs001529.v1.p1 [17]. (3) Zhang, F. et al. dataset: Single SF were sorted (CD45−CD31−PDPN+), libraries produced with CEL-Seq2 protocol and sequenced on the Illumina HiSeq 2500 platform. The raw single-cell RNA-seq data are deposited in dbGaP (dbGaP Study Accession: phs001457.v1.p1) [19].

During the first step of the analysis, human genes were converted into mouse homologs using the Ensembl Biomart and MGI database, leading to the final set of 17,594 homologous pairs. Regarding the cells that were used, from the mouse dataset, only the cells originating from the pooled *hTNFtg* samples (3051 cells) were processed, while from the three human datasets, only the cells originating from RA patients (24,042 cells). Consequently, the integration strategy described in [23] was followed through the Seurat package. More specifically, all four datasets were processed by applying normalization and most-variable-gene detection using the function normalizeData with default settings and FindVariableFeatures (method set to vst and number of variable features to 2000), respectively. Anchors between all datasets were identified using the function FindIntegrationAnchors with dimension parameter set to 30, and then, these anchors were utilized to integrate the four datasets together using the function IntegrateData. The final object containing all cells from both species was processed in a standard way, performing the steps of dimensionality reduction, clustering, and marker gene detection. The integrated clusters were defined after using the FindClusters function with a 0.3 resolution. Finally, marker gene detection was performed by using findAllMarkers function with the following thresholds: *p*-value < 0.01 and avgLFC ≥ 0.25. Regarding the functional enrichment analysis, the upregulated genes of human and mouse datasets were used as an input for Metascape [33], significant terms and pathways (*p*-value < 0.05) were used to assess the similarities and differences across the datasets. (For all the comparisons between humans and mice described above, the final integrated object was split into two, one containing all human cells from the three different datasets and another containing all mouse cells from pooled *hTNFtg* samples.)

### Integration of WT,* hTNFtg*, and STIA datasets

We used a publicly available sc dataset from serum transfer-induced arthritis (STIA) model deposited in the Gene Expression Omnibus (GEO) (accession code GSE129087) [18]. For the generation of the STIA dataset, CD45-ve live synovial cells from the hind limb joints were isolated and sort purified at day 9 (*n* = 3 biological replicates, each comprised of cells from the joints of three animals) and captured with the 10X Genomics Chromium system [18].

The integration strategy that has been described before was followed employing the Seurat package. More specifically WT, *hTNFtg*, and STIA datasets were processed by applying normalization and most-variable-genes detection using the function normalizeData with default settings and FindVariableFeatures (method set to vst and number of variable features to 2000) respectively. Anchors between samples were identified using the function FindIntegrationAnchors with dimensions parameter set to 30, and then these anchors were utilized to integrate all the samples together using the function IntegrateData. The final object, containing all cells from the control and both arthritic models, was processed in a standard way, performing the steps of dimensionality reduction and clustering. The integrated clusters were defined after using the FindClusters function with a 0.4 resolution. Finally, the marker genes displayed in (Additional file 1: Fig. S6E) were selected from the supplementary material of *hTNFtg* and STIA analyses (Additional file 2: Table S1 from the current manuscript and extended data Fig. 6 from [18]).

### Isolation of RNA and bulk 3′ RNA sequencing

Mice from both sexes were included for the generation of the RNA samples. Three individual RNA samples per condition were prepared by sorted ankle joint SFs (sublining/Pdpn+ Thy1+ and lining/Pdpn+ Thy1−) of healthy *Col6a1Cre ROSA26*^*mT/mG*^ (4 weeks of age, 1–2 mice/sample) [7, 34] and *hTNFtg Col6a1Cre ROSA26*^*mT/mG*^ mice (4 and 8 weeks of age, ankle SFs from 1–2 mice/sample) using the RNeasy mini or micro kit (QIAGEN), according to the manufacturer’s instructions. The quantity and quality of RNA samples were analyzed using Agilent RNA 6000 Nano kit with the bioanalyzer from Agilent. RNA samples with RNA integrity number (RIN) > 7 were used for library construction using the 3′ mRNA-Seq Library Prep Kit Protocol for Ion Torrent (QuantSeq-LEXOGEN™) according to the manufacturer’s instructions. DNA High Sensitivity Kit in the bioanalyzer was used to assess the quantity and quality of libraries, according to the manufacturer’s instructions (Agilent). Libraries were then pooled and templated using the Ion PI™ IC 200 Kit (Thermo Fisher Scientific) on an Ion Proton Chef Instrument or Ion One Touch System. Sequencing was performed using the Ion PI™ Sequencing 200 V3 Kit and Ion Proton PI™ V2 chips (Thermo Fisher Scientific) on an Ion ProtonTM System, according to the manufacturer’s instructions.

### Computational analysis of bulk RNA sequencing data

The quality of the FASTQ files was assessed with the fastqc software (Andrews, S. (2010). FastQC: a quality control tool for high throughput sequence data [Online]. Available online at: http://www.bioinformatics.babraham.ac.uk/projects/fastqc/). Reads were aligned to the mm10 genome were performed with the Hisat2 aligner. FeatureCounts [35] was utilized for the step of read summarization at the gene level. Differential expression analysis was conducted by DESeq2 [36]. For each contrast, differentially expressed genes were defined by applying the following thresholds |Log2FC| > 0.58 and *p*-value < 0.05.

### Droplet-based single-cell ATAC sequencing

Single-cell assay for transposase-accessible chromatin using sequencing (scATAC-seq) protocol was performed according to 10X Genomics instructions. Samples were obtained from mice of both sexes to avoid sex bias effect in downstream analyses. The ankle joints were dissected from WT mice at the age of 4 weeks (*n* = 3) and *hTNFtg*, at the age of 4 weeks (*n* = 3) and at the age of 8 weeks (*n* = 3). Briefly, after sorting of synovial fibroblasts (see the scRNA-seq protocol for details) and nuclei isolation, the nuclei were resuspended in 1× Diluted Nuclei Buffer (10X Genomics). About 4600 nuclei were added in each transposition reaction, aiming for a targeted nuclei recovery of 3000 nuclei. Transposition was performed at 37°C for 60 min. Generation of Gel beads in EMulsions (GEMs) using Chromium Controller (10X Genomics), was followed by GEM incubation and cleanup, based on 10X Genomics recommendations. Amplification of libraries was performed in a Veriti Thermal Cycler (Thermo Fisher) programmed at 98°C for 45 s followed by 12 cycles of (98°C for 15 s, 67°C for 30 s, 72°C for 20 s), 72 °C for 1 min and hold at 4 °C. In turn, libraries were double-sided size selected using SPRI select reagent (Beckman Coulter) according to 10X Genomics recommendations. Before multiplexing, libraries were assayed on Bioanalyzer High Sensitivity DNA ChIP (Agilent), for quality check and determination of fragment size. Quantification of libraries was performed using Qubit dsDNA HS Assay Kit (Thermo Fisher, Cat. No Q32851). Next-generation sequencing was performed at EMBL-Genecore (Heidelberg), using the NextSeq 500 platform for paired-end 75-bp reads.

### Computational analysis of single-cell ATAC-seq

**Fig. 1:**
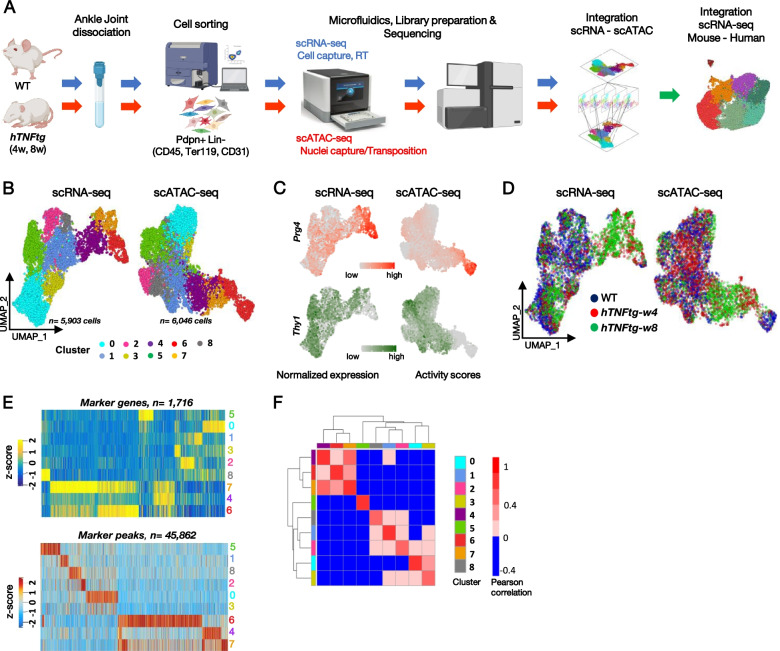
Multiomic transcriptional and epigenetic single-cell analysis of SFs. **A** Schematic representation of the experimental workflow. We collected ankle synovial tissue from wt and *hTNFtg* mice, enzymatically disaggregated the tissue, and sorted the cells into one gate representing fibroblasts (CD45−, Ter119−, CD31−, Pdpn+). We profiled the cells with both sc 3′ RNA-seq and ATAC-seq using 10X technology and performed scRNA-seq, scATAC-seq, and cross-species integrative analyses with publicly available human RA datasets. **B** High-quality filtered synovial fibroblasts (*n* = 5903 for the scRNA-seq and *n* = 6046 for the sc-ATAC-seq) projected in UMAP space and colored by cluster assignment. **C** Feature plots on the UMAP embeddings of the SFs shown in **B**, displaying normalized expression values (for scRNA-seq) and gene activity scores (for scATAC-seq) for Prg4 and Thy1 genes. **D** Similar to **B**, but cells are colored by the sample of origin. **E** scRNA-seq heatmap showing the average scaled expression values for the upregulated genes of each subpopulation (upper panel) and scATAC-seq heatmap of differentially upregulated accessible peaks (lower panel). **F** Pearson correlation of scaled expression values (RNA) and activity scores (ATAC), followed by hierarchical clustering, for the most variable genes identified in the scRNA-seq analysis

The analysis of scATAC-seq datasets was conducted by using the ArchR suite [37]. Reads were counted across the genome, using 500-bp bins (tiles) to generate a genome-wide tile-count-matrix. Epigenetic maps of sorted SFs nuclei were obtained for 6679 single nuclei. Latent semantic indexing (LSI) [38, 39], Louvain clustering, and UMAP dimensionality reduction were applied as described above (see the “Computational analysis of single-cell RNA sequencing data” section). Gene activity scores were computed as the summed local accessibility of promoter-associated count-tiles in the proximity of each gene, using a distance-weighted accessibility model. In particular, count-tiles within 100,000 bp of a gene promoter were aggregated using a distance weight e(−abs (distance)/5000) + e−1). To account for gene length biases, an additional normalization was applied (multiplication by 1/gene size, scaled linearly from 1 to 5). Finally, the above-weighted sum was multiplied by the aggregated Tn5 insertions in each tile. Gene scores were then scaled to 10,000 counts and log2-normalized. To enhance the visual interpretation of gene activity scores, a smoothing was applied using the MAGIC algorithm [40]. To assign scATAC-seq cluster identity, gene activity scores and scRNA-seq gene expression were directly aligned between the two modalities [23], by first applying an unconstrained integration to gain prior cluster identity knowledge, that was in turn used as a guide for a more refined constrained integration [37]. This procedure grouped cells into 5 major clusters, corresponding to the previously annotated cell types described above (synovial fibroblasts, osteoblasts, chondrocytes, myoblasts/myocytes, and vascular cells, Additional file 1: Fig. S3). All non-fibroblast cells were excluded from the rest of the analysis, resulting in a total of 6,046 SF cells that were re-analyzed in the same fashion. The integration process between scATAC-seq and scRNA-seq SFs labeled the scATAC-seq cells according to 9 SF subpopulations (see above) that were visualized in UMAP space (Fig. 1; Additional file 1: Fig. S3). To identify a robust merged peak set along the SF subpopulations, MACS2 [41] was applied at two separate pseudo-bulk replicates [37]. Next, iterative overlap peak merging [42] was applied at the level of the pseudo-bulk replicates (per subpopulation), and subsequently at the level of SF subpopulations across the whole dataset, to form a single merged peak set of 158,713 regions with a fixed length of 500 bp. In turn, peaks were annotated according to their respective genomic position (promoter, intronic, exonic, distal). Using the unified peak set, differential accessibility analysis between cells was performed to identify cluster-specific and condition-specific marker peaks (|Log2FC| > 0.58 and *p*-value < 0.01). Marker peaks were further analyzed using motif enrichment analysis (CIS-BP database), to gain cluster-specific and sample-specific marker motifs ( |Log2FC| > 0.58 and *p*-value < 0.05). To further gain enriched motifs in single-cell resolution, chromVar analysis was conducted [43]. Consequently, to identify “positive TF regulators” in SF subpopulations, TF motif accessibility was correlated with integrated TF gene expression across cells, keeping all TFs with Pearson *r*^2^ > 0.5 and *p*-adjusted value < 0.05, resulting in 30 positive regulators. Finally, to identify the underlying GRNs, peak to gene linkages were called using correlation analysis between enhancer peak accessibility and integrated gene expression (see addPeak2GeneLinks() function of ArchR R package) [37]. All links between genes and accessible regions with an annotated TF motif were marked as putative regulatory links between the respective TF and gene. Subsequently, all putative regulatory links were filtered to only keep genes that are upregulated in *hTNFtg* samples, as also peaks with increased accessibility in the disease samples.

## Results

### Multi sc-omic analysis of* hTNFtg *mouse model of chronic inflammatory polyarthritis

To characterize disease progression and pinpoint what differentiates homeostasis from pathogenesis at the level of SF subpopulations in synovium, we integrated sc transcriptomic and chromatin accessibility profiles (Fig. 1A). We included cells from healthy tissue (WT, 4 weeks of age (*n* = 3)), *hTNFtg* mice at an early disease stage displaying synovial inflammation (*hTNFtg-w4*, 4 weeks of age (*n* = 3)), and at an established pathological stage displaying pannus formation, inflammation, cartilage, and bone damage (*hTNFtg-w8*, 8 weeks of age (*n* = 3)) (Additional file 1: Fig. S1A). Synovial non-hemopoietic, non-endothelial, and non-erythroid cells were sorted (CD45^−^, CD31^−^, Ter119^−^, Pdpn^+^) and used to generate scRNA-seq libraries (10X Genomics, reconstitution of total 6667 cells) (Fig. 1A and Additional file 1: Fig. S1B, C). In parallel, the same cell isolation protocol was employed to perform single-cell transposase-accessible chromatin using sequencing from nuclei (scATAC-seq, 10X Genomics, reconstitution of 6679 single cells/nuclei). Healthy and *hTNFtg* cells were pooled in each experimental modality, to create a common baseline between homeostatic and pathogenic conditions.

Upon filtering out the non-fibroblast cells based on well-known transcriptomic markers as previously reported [18] (Additional file 1: Fig. S1C, D), we additionally refined the scATAC-seq cluster annotation using canonical correlation analysis (CCA) to enable the matching of scRNA-seq and scATAC-seq cluster identities (Fig. 1F and the “Methods” section). We focused on the 5903 and 6046 cells/nuclei presenting SFs characteristics in scRNA-seq and scATAC-seq respectively (Fig. 1B). Sub-clustering analysis of SF-specific molecular maps resolved nine fibroblastic clusters (Fig. 1B and Additional File 1: Fig. S1F, G). Using as a proxy the classical markers Prg4 and Thy1 [16, 18, 19], we observed a compartmentalization of Prg4^high^ (LSFs) vs Thy1+ SFs (SLSFs) (Fig. 1B, C). We also noted that two clusters (4 and 7) are mainly present in the disease (*hTNFtg-*w4,8) and they expressed both *Thy1* and *Prg4* genes (Fig. 1C, D). Graph-based clustering followed by differential expression analysis (DEA) at the single-cell RNA-seq level revealed 1716 marker genes (i.e., upregulated in at least one cluster vs the others), while the differential peak accessibility analysis at the single-cell ATAC-seq level identified 45,862 marker peaks (i.e., with significantly increased accessibility in at least one SF subpopulation compared to the others). Inspection of the aforementioned marker genes and peaks revealed cell specificity and shared patterns both at transcriptional and chromatin levels (Fig. 1E). In fact, high correlation coefficient scores between gene expression and chromatin accessibility were observed not only within clusters, but also across clusters and suggested some architectural/functional overlap among SLSFs and among LSFs and SF clusters 4 and 7 (Fig. 1F). Therefore, our combined-omics approach deconvolved SF varieties with specific patterns of gene expression and associated chromatin accessibility signatures, which may be used to further characterize RA molecular markers and determine the underlying gene regulatory networks driving its pathophysiology (see below).

### High-resolution maps of transcription regulation in homeostatic joints

**Fig. 2:**
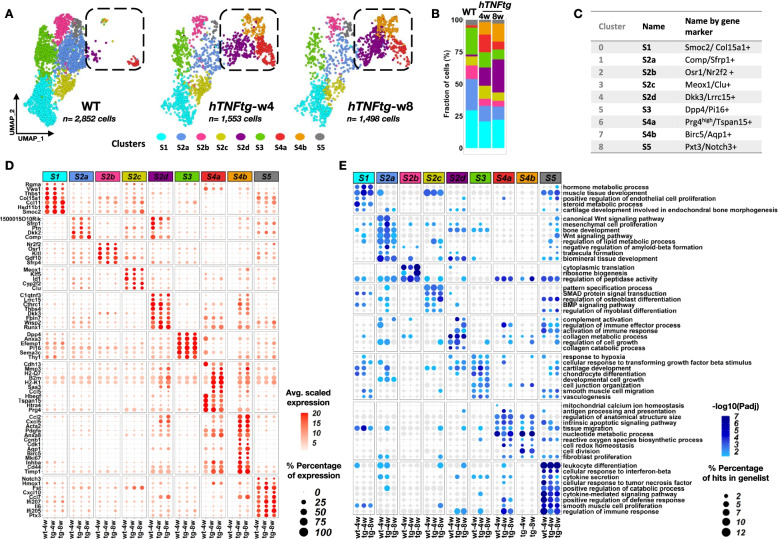
Structural remodeling of the synovial mesenchyme in the *hTNFtg* arthritic joint. **A** UMAP representation of SFs across the three different samples (WT, *hTNFtg*/4 weeks, and *hTNFtg*/8 weeks as indicated). The cells are colored by cluster identities, and the marked areas highlight the structural dynamic changes of the intermediate and lining subpopulations in RA evolution/during disease progression. **B** Stacked bar charts showing the relative abundances (%) of clusters across samples (WT, *hTNFtg* 4 and 8 weeks). **C** Table summarizing the cluster numbers, simplified names, and marker genes. **D** Dot plot of the cluster marker genes. The color of the dot shows the intensity of expression while the size denotes the percentage of cells expressing the gene in each cluster and condition (WT: wt-4w; *hTNFtg*/4: tg-4w; *hTNFtg*/8: tg-8w). **E** Functional enrichment analysis indicating the enriched biological processes for each cluster across samples. Significance is noted by color, and the percentage of cluster marker genes included in each term is noted by size

To evaluate the qualitative and quantitative difference of each cluster per condition, we distributed and visualized the cells from each sample in individual UMAPs (Fig. 2A, B). We further annotated the nine SF clusters by taking into account the inter-cluster and intra-cluster analysis of DE genes (Additional file 3: Table S2) and current literature regarding fibroblast profiles [18–20, 44]. We named the clusters employing gene names: Smoc2/Col15a1+ (cluster 0/S1), Comp/Sfrp1+ (cluster 1/S2a), Osr1/Nr2f2+ (cluster 2/S2b), Meox1/Clu+ (cluster 3/S2c), Dkk3/Lrrc15+ (cluster 4/S2d), Dpp4/Pi16+ (Cluster 5/S3), Prg4^high^/Tspan15+ (Cluster 6/S4a), Birc5/Aqp1+ (cluster 7/ S4b), and Pxt3/Notch3+ (Cluster 8/S5). However, for reasons of simplicity, from now on, we use the cluster acronyms (S1, S2a,b,c,d, S3, S4a,b and S5) (Fig. 2C).

We first characterized RNA expression specificities in healthy homeostatic joints by looking at cluster-specific upregulated genes in WT SFs independently (Fig. 2B, C, D and Additional file 2: Table S1). The S4a SFs were devoid of Thy1 expression, and they expressed high levels of *Prg4* (Prg4^high^, Additional file 1: Fig. S2A) along with other genes previously reported as markers of the lining phenotype (LSFs), such as *Tspan15*, *Hbegf*, and *Htra4* [18, 19] (Fig. 2D). In WT joints, these LSFs were clearly demarcated from the sublining cells (Thy1+, Prg4^low/−^) because of the very limited number of S2d and S4b cells (Thy1+, Prg4^high^) (Fig. 2A, B). By estimating the percentage of Thy1-positive, Prg4-positive, and double-positive SF cells in each cluster per sample, we observed that in WT tissues, *Thy1* and *Prg4* are mainly expressed on mutually exclusive SF subsets while *in hTNFtg*, *Thy1*, and *Prg4* exhibit more coexpression specially in the disease-enriched clusters S2d and S4b (Additional File 1: Fig. S2A). Functional enrichment analysis revealed that, in contrast to the reported destructive profile of the lining cluster in arthritic disease [18], in normal conditions, LSFs tend to preserve tissue homeostasis by uniquely performing the negative regulation of oxidative stress-induced cell death, as well as the homeostasis of mitochondrial calcium, a fundamental signaling modulator [45] (Fig. 2E, Additional file 1: Fig. S3, Additional file 3:Table S2).

Regarding Thy1^+^ SLSF populations, we find that S1 transcriptional state is marked by the expression of *Smoc2*, *Thbs1*, *Vwa1*, and *Col15a1* genes that encode matricellular proteins and the BMP co-receptor *Rgma* (Fig. 2D and Additional file 2: Table S1, Additional file 3: Table S2), along with BMP/SMAD signaling pathways detected in the GO enrichment analysis (Fig. 2E and Additional file 3: Table S2). The expression of genes associated with steroid metabolism, including the cortisone-conversion enzyme *Hsd11d1* [46], provides S1 cells an anti-inflammatory role.

S2 SF subtypes are characterized by common (*Comp*, *Ptn*, *Gdf10*) and divergent marker genes and functions (Fig. 2D, E and Additional file 2: Table S1, Additional file 3: Table S2). In particular, the S2a population is defined by the high expression of WNT modulators *Dkk2* and *Sfrp1*, in accord with the GO enrichment in WNT-mediated responses, TGF activity, and osteogenesis. In addition, the specific expression of *Ecrg4* (1500015O10Rik) gene indicates a role of S2a in regulating tissue repair processes (wound healing) [47]. In S2b, gene expression is linked to joint morphogenesis and reparative processes; e.g., *Osr1* regulates *Prg4* [48] and plays a pivotal role in fibroblast differentiation [49]. Moreover, *Nr2f2* (COUP-TFII) marker gene is implicated in cell fate decisions of stem cells [50]. S2c SF state is characterized by BMP signaling pathway activation and osteoblast and myoblast differentiation. Characteristic gene expression involves the *Klf5*, *Clu*, Id*1*, and *Meox1* genes.

The gene expression signature of S3 indicates that these SFs drive processes relative to vasculogenesis and regulation of type 2 immune responses and myeloid lineage differentiation and homeostasis. S3 SFs are characterized by the expression of *Pi16* which functions in pain and fibroblast/endothelial crosstalk [51], the physiological vascular normalizing modulators *Sema3c* [52] and *Efemp1* [53, 54], and the glucose and immune regulator *Dpp4* [55] (Fig. 2D, E, Additional file 1: Fig. S4 and Additional file 2: Table S1, Additional file 3: Table S2).

Finally, S5 cells show activation of cytokines and chemokine pathways (*Ccl7*, *Cxcl10*, *IL6*, and *Ptx3*) and are associated with immune-regulatory functions including response to IFN-beta/gamma and LIF, indicating a strong immunoregulatory role in the synovial membrane under healthy conditions. Notably, *Notch3*, a gene recently highlighted for its role in driving SF identity in the perivascular/sublining layer of arthritic synovium [20], is also expressed in normal conditions mainly in cluster S5 (Fig. 2D, E and Additional file 2: Table S1, Additional file 3: Table S2).

Overall, the analysis of SFs in naïve conditions highlights a previously underexplored functional diversity underlying the homeostasis of the synovial membrane.

### Development of inflammatory arthritis associates with the transcriptional remodeling of SF populations and functions

We next sought to dissect the processes underlying the appearance and maintenance of TNF-induced pathological states of SFs. The differential abundance analysis of *hTNFtg* compared to WT SFs showed not only aberrations in SF clusters but also revealed disease-enriched subpopulations (Fig. 2A, B). The proportion of S2d and S4b cells gradually increased from almost undetectable levels (2 and 0.17%) in healthy conditions to 25 and 14% in the *hTNFtg* joints of established disease (8 weeks old), respectively (Fig. 2A, B). Correlation analysis on the most variable genes (MVGs) of SF clusters highlighted a striking overlap in the transcriptional profiles of the Prg4^high^ S4a and the intermediate S4b and S2d SF subpopulations (Additional file 1: Fig. S2A), which was already suggested from the patterns of selected representative marker genes and GOs (see Fig. 2D, E). Correlation scores were higher between *hTNFtg* cells indicating an acute and stable change in the particular SFs expression signatures after the onset of arthritis (Additional file 1: Fig. S2A). Besides the original observation that *Thy1* and *Prg4* exhibit more coexpression compared to WT tissues (Figs. 1C, D and 2B, C), we also observed a striking overlap in the transcriptional profiles of the Prg4^high^ S4a SFs and the intermediate S4b and S2d SFs (Additional file 1: Fig. S2B and Fig. 2D, E). The gain in the number of these “intermediate” and lining S4a cells was offset by the shrinkage in the proportion of the number of other cell types S2a, S2b, S2c, S3, and, to a lesser degree, S1 and S5 (Fig. 2B, C); these “shrunk” clusters showed a more homogenous signature and less DE genes between WT and *hTNFtg*, indicating common and stable functions in healthy and disease joints (Additional file 1: Fig. S2A, B and Additional file 2: Table S1). However, besides some very unique functions in each stage of disease for each cluster (Additional file 1: Fig. S5-6, Additional file 3: Table S2), we also noted an early and stable gain of some important disease-related processes. According to their de novo transcriptome changes during disease, the S1(Smoc2/Col15a1+) SFs positively regulate fibroblast migration and apoptotic processes. The S2a(Comp/Sfrp1+), S2c(Meox1/Clu+), S3(Dpp4/Pi16+), and S5(Ptx3/Notch3+) SFs exhibit phosphatidylinositol 3-kinase signaling. The S2a (Comp/Sfrp1+) SFs further show the positive regulation of developmental growth, while S2c(Meox1/Clu+) and S5(Ptx3/Notch3+) SFs participate in osteoclast differentiation. S3(Dpp4/Pi16+) SFs engage in blood vessel remodeling throughout arthritic stages. The S5(Ptx3/Notch3+) SFs regulate monocyte differentiation and uniquely present activation of protein kinase B activity, positive regulation of stress-activated MAPK cascade, and positive regulation of response to hepatocyte growth factor during arthritic disease (Fig. 2E, Additional file 1: Fig. S5A, Additional file 3: Table S2). On the other hand, loss of functions per shrunk cluster during the progression of arthritis is highlighted in S1(Smoc2/Col15a1+) SFs, by, e.g., the disappearance of characteristic steroid biosynthetic process, BMP signaling, and chondrogenesis, while in S5(Ptx3/Notch3+) SFs, the regulation of several cytokine responses and tissue regeneration were also lost. Similarly, in S2a SFs, the functions of Wnt regulation, epithelial-to-mesenchymal transition, and androgen receptor signaling are gradually reduced during disease. In S3(Dpp4/Pi16+) SFs, the responses to hypoxia, the regulation of TGFβR signaling, the development of cartilage, and the type 2 immune responses are also absent from established disease. All these discrepancies (loss or gain of functions) likely reflect the hypo-populated however arthritis-reoriented SF states (Fig. 2E, Additional file 1: Fig. S5B, Additional file 3: Table S2).

The “expanding” S2d(Dkk3/Lrrc15+) SFs express highly important genes for joint pathology (Fig. 2D, E) including the ECM component *Fbln7* [56], the matricellular protein *Thbs4*, the vascular remodeler *Cthrc1* which has also been proposed as a marker for embryonic progenitors of SFs, fibrocartilage cells of the enthesis [57], and fibrotic lung fibroblasts [58]. The expression of *Dkk3* associates the murine S2d transcriptional state with the previously described human SC-F3 (DKK3+) SF cluster [19]. The S2d SFs also express *Lrrc15*, a recently identified marker for cancer-associated fibroblasts (CAFs) and activated fibroblasts [44, 59] and the TF Runx1. In accord, we find multiple biological processes including regulation of immune and redox response, cell fate determination, and ECM remodeling, which indicate a multi-potent transcriptional signature S2d SFs.

The S4b(Birc5/Aqp1+) SFs, further to high Prg4/Thy1 marker genes, also express *Mki67*, *Pdgfa*, *Birc5*, *Aqp1*, *Acta2*, the *C1qtnf3* adipokine, and other chemokines such as *Cxcl5*, as well as several adhesion molecules. The functional annotation related to increased proliferating capacity, adhesion, and peptidase activity reinforcing the idea that these cells actively contribute to the inflammatory process in arthritis (Fig. 2D, E; Additional file 2: Table S1, Additional file 3: Table S2).

**Fig. 3:**
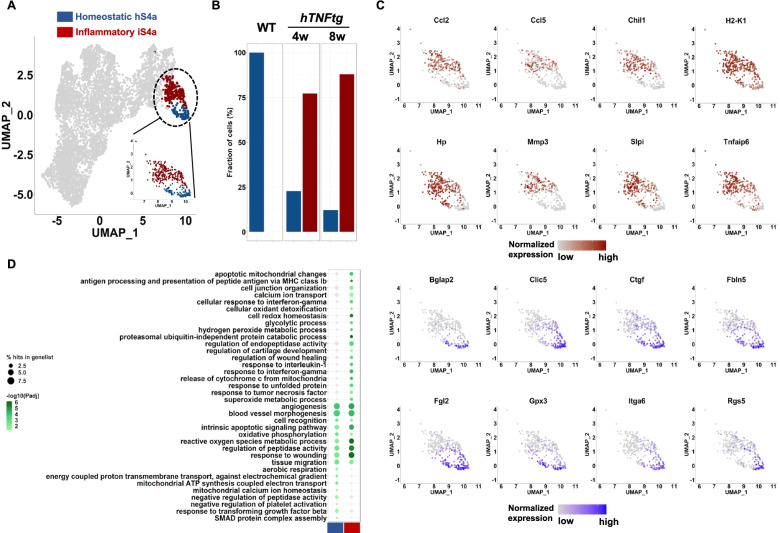
Sub-clustering analysis of S4a SFs revealed a homeostatic and an inflammatory state of LSFs. **A** UMAP representation of the identified lining sub-clusters. Cells belonging to the homeostatic group (hS4a) are colored in blue, while cells belonging to the inflammatory group (iS4a) are colored in red. **C** Barplot showing the relative abundances of cells in each sub-cluster in healthy conditions and during disease progression. **C** Feature plots of selected genes showing differential patterns of expression in the two sub-clusters. **D** Dot plot of shared and specific enriched GO biological processes in the two sub-clusters.

During TNF-mediated arthritis, the S4a(Prg4^high^/Tspan15+) LSFs preserve some of their homeostatic marker gene identity, but also show an expansion in the diversity of their transcriptome, indicating that their reparative functions might be affected after disease onset. We detected markers of inflammatory response (*Ccl2*, *Ccl5*, *Hmox1 Saa3*), class I antigen presentation (*H2-K1*, *B2m*, *H2-Q7*), and ECM remodeling (*Mmp3*, *Timp1*, *Cd44*) (Fig. 2D, E), in agreement with previous reports on arthritic LSFs [18, 19]. The expansion of LSFs is also accompanied with some loss of homeostatic functions during disease progression, such as ER calcium homeostasis and response to oxygen levels (Additional file 1: Fig. S5B). Notably, a meticulous sub-clustering analysis of the S4a cluster confirmed the presence of two groups of cells (subclusters hS4a (homeostatic) and iS4a (inflammatory)) (Fig. 3A, B), where the emergence and expansion of the inflammatory state iS4a dominate during disease, at the expense of homeostatic state hS4a (Fig. 3C, D and Additional file 4: Table S3).

Interestingly, when we integrated our normal and *hTNFtg* dataset with the respective data derived from a previous study on mouse acute inflammatory arthritis (STIA), we noted a similar pattern of both expansion and shrinkage of SF clusters compared to normal SF statuses (Additional file 1: Fig. S6A-D).

We validated the expression of scRNA-seq-derived markers in murine joints by employing spatial detection by multicolor immunofluorescence. Clu (S2c, Meox1/Clu+ SFs) and Sema3c expression (S3, Dpp4/Pi16+ SFs) are sparsely distributed in the sublining compartment of healthy joints. In the *hTNFtg* joints, their expression is scattered throughout the inflammatory sublining synovium (Additional file 1: Fig. S7A). S2a/b-associated marker Gdf10 and the S2a(Comp/Sfrp1+) marker Comp are detected in Thy1+ cells, directly adjacent to the lining outermost cellular layer and closer to the cartilage (Additional file 1: Fig. S7A,B). Consistent with the scRNA-seq results, the S5(Ptx3/Notch3+) marker Notch3 is restricted to a smaller Thy1^+^ SLSF subpopulation, colocalizing around the vascular cells (CD31+) in WT joints. The Notch3 expression remains limited to perivascular areas in the *hTNFtg* joint (Additional file 1: Fig. S7A). Interestingly, Notch3+ cells (S5− Ptx3/Notch3+ cluster) and Gdf10+ and Smoc+ cells (S2a-Comp/Sfrp1+ and S2b-Osr1/Nr2f2+ clusters) are excluded from the interface of pannus/cartilage-bone junction (Additional file 1: Fig. S7A,B).

A specific spatial trend was identified for Dkk3/Lrrc15+ (S2d) and Birc5/Aqp1+ (S4b) intermediate SFs. As indicated by the transcriptomic analysis, their marker a-SMA (Acta2) is absent from the healthy synovium, and it is exclusively detected in the pericytes of WT joints. Consistent with the RNA expression, CD44 expression is present in both the sublining and lining compartments of healthy synovia (Additional file 1: Fig S7B). However, in disease, a-SMA, CD44, Prg4, Dkk3, Mik63, and Runx1 proteins are detected at the interface of the invasive synovial tissue and the articular bone, indicating a distinct localization of the intermediate S2d(Dkk3+/Lrrc15+) and S4b(Birc5/Aqp1+) clusters. Notably, their distribution is evident at both sublining and lining compartments. Finally, we validate in situ the expansion of high Prg4-expressing SFs (S4 clusters) in the diseased joints (Additional file 1: Fig. S7C).

Collectively, all the above findings establish detailed molecular, functional, and anatomical maps outlining the dynamic and diverse effects of the development and progression of the pathogenic SF states in arthritic mice.

### Bulk markers of the inflammatory expansion of SFs in TNF-mediated arthritis

Taking advantage of our scRNA-seq results, we looked for reliable arthritic marker gene expression in whole tissues. We performed bulk RNA-seq on sorted LSFs and SLSFs. LSFs (CD31−, CD45−, Ter119−, CD90−, Pdpn+) and SLSFs (CD31−, CD45−, Ter119−, CD90+, Pdpn+) derived from naive, and 4w and 8w diseased mice—additionally marked by a SF-specific GFP marker (see the “Methods” section)—showed a clear separation (Additional file 1: Fig. S8A). Bulk RNA-seq DEA and comparison with DEA performed on scRNA-seq data (see above) revealed commonly identified genes and confirmed that more differences in gene expression is detected between lining (L) and sublining (SL) SFs in healthy animals (WT) compared to *hTNFtg* counterparts (Additional file 1: Fig. S8A-C and Additional file 5: Table S4), further suggesting that SFs tend to lose their sharp healthy bi-modal (lining vs sublining or Prg4 vs Thy1 compartmentalization) character in arthritic tissues (see Fig. 1C, D, Additional file 1: Fig. S2A). We confirmed this by calculating bulk fold changes (FCs) between LSF and SLSF and show how genes we established as S4a marker genes fit with a LSF signature in bulk data. In contrast, S2d and S4b genes are more equally expressed in both states, and the remaining clusters tend to be defined by genes upregulated in the SLSF state (Additional file 1: Fig. S8D). Corroboratively, we found that more genes of sc cluster S4a are detected as bulk LSF markers, more genes representative of S5, S2b, and S2b are bulk markers of SLSFs, while a balanced number of S2d and S4b markers are found in LSF and SLFs bulk DE gene (DEG) lists (Additional file 1: Fig. S8E). Interestingly the genes co-differentially expressed in sc and bulk (see Additional file 1: Fig. S8C) constitute a robust list of marker genes characterizing SL and L SFs and is also highlighting genes marking the intermediate (I) state (Additional file 1: Fig. S8F, Additional file 5: Table S4). Focusing on genes showing significant FC between LSF and SLSF in healthy (WT) or pathogenic (*hTNFtg-4w* and *hTNFtg-8w*) joints in the bulk data (Additional file 1: Fig. S8G), we propose potential diagnostic genes, which may be used as a panel to test disease status by performing qPCR on sorted SFs obtained from biopsies.

### Development of arthritic pathology depends on activated epigenomic states and distinct gene regulatory networks in SFs

**Fig. 4:**
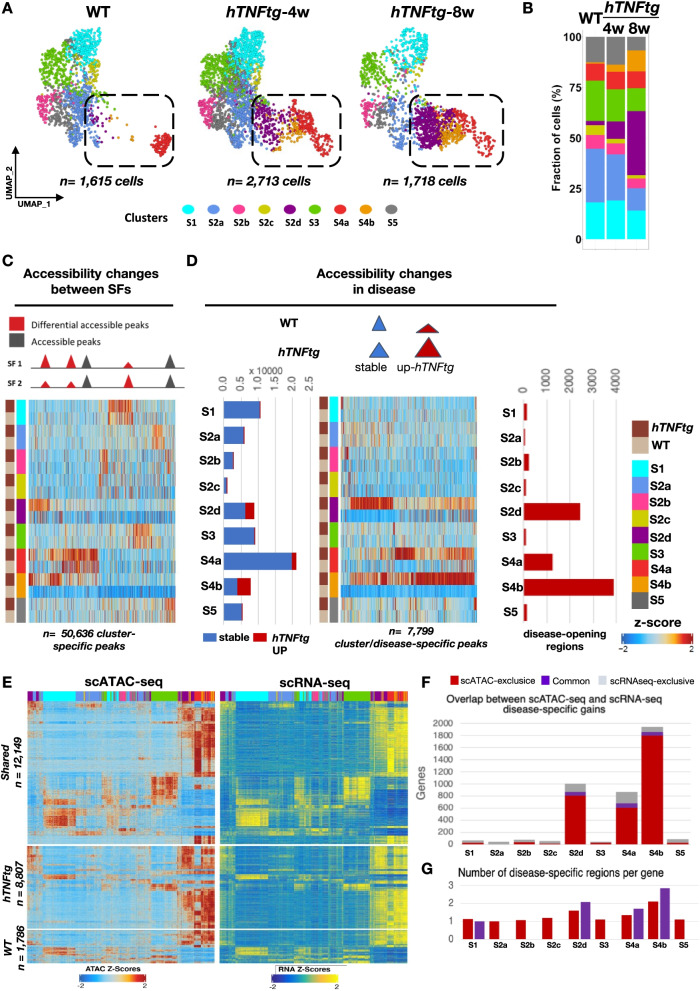
scATAC-seq recapitulates the structural remodeling of the synovial mesenchyme in the *hTNFtg* arthritic joint. **A** UMAP representation of SFs across the three different samples (WT, *hTNFtg*/4 weeks, and *hTNFtg*/ 8 weeks as indicated). Cells are colored by cluster identities, and the marked areas highlight the structural dynamic changes of the intermediate and lining subpopulations during disease progression. **B** Stacked bar charts showing relative abundances (%) of clusters across samples (WT, *hTNFtg* 4 and 8 weeks). **C** Upper panel: schematic representation of the marker peak detection procedure. Lower panel: heatmap showing the column *Z*-score of normalized accessibility of 50,636 marker peaks, across SF subpopulations and disease states (WT, *hTNFtg*). **D** Upper panel: schematic representation of the disease-specific marker peak definition. Left panel: stacked bar chart depicts the proportions of stable and *hTNFtg*-specific marker peaks. Center panel: heatmap showing the column *Z*-score of normalized accessibility of 7,799 disease-specific marker peaks (WT, *hTNFtg*), across SF subpopulations. Right panel: bar chart depicts the distribution of regions with an increased opening in disease vs WT across clusters. **E** Heatmap showing the column *Z*-score of normalized accessibility and integrated gene expression of 52,133 peak-to-gene links across WT and *hTNFtg* SF subpopulations. Upper panel: peak-to-gene links that are shared between conditions. Middle panel: peak-to-gene links that are unique to *hTNFtg* cells. Lower panel: peak-to-gene links that are unique to WT cells. **F** Stacked bar chart depicting the number of disease-specific marker genes (described in **E**, middle part) exclusively found in scATAC-seq data (red), shared between modalities (purple), and exclusively found in scRNA-seq data (gray). **G** Bar chart depicting the number of regions per gene with gains in accessibility detected in disease.

To identify the pathogenic molecular master switches that remain repressed in healthy joints and are activated in arthritis, we also analyzed scATAC-seq data to find condition- and cell type-specific chromatin signatures and explored what TF and target genes are controlled at the epigenomic level (see the “Methods” section). Accessible chromatin patterns recapitulated the significant expansion of the SFs subpopulations S2d(Dkk3/Lrrc15+) and S4b(Birc5/Aqp1+) upon disease progression (Fig. 4A, B). We performed a two-level analysis of open chromatin regions (OCRs). To avoid a potential pitfall due to the very low number of S2d(Dkk3/Lrrc15+) and S4b(Birc5/Aqp1+) cells in WT and given the inherent sparsity of scATAC-seq, we determined OCRs in the joint dataset (WT with *hTNFtg* cells). We first identified differential accessibility patterns across the union of all accessible regions (*n* = 158,713), and we found 50,636 peaks (Fig. 4C) showing SF subtype-specific patterns of accessibility. In particular, more regions defining intermediate (S2d, S4b) and lining (S4a, Prg4^high^/Tspan15+) cells were evident (Fig. 4C, D). Second, we highlighted striking gains in DNA accessibility upon disease (more accessibility in *hTNFtg* than in WT) at 27.9 and 49.8% of cluster-specific loci for S2d and S4b (Fig. 4D). By determining peak-to-gene linkages (Fig. 4E and the “Methods” section), we established that many gene regulatory links (genes associated with given OCRs) appeared conserved across conditions (Fig. 4E) and that most genes did not display noticeable changes at the chromatin level, in agreement with the observation that a large majority of the OCRs remain stable (see Fig. 4D, left panel). In contrast, for the OCRs that change upon disease, we reveal 1,786 and 8,807 region-to-gene associations that distinguish healthy and *hTNFtg* SFs (Fig. 4E). Importantly, many of the upregulated genes in intermediate cells in disease show a parallel gain in accessibility at the linked open regions. For example, up to 40% of the genes upregulated in *hTNFtg* SFs, according to scRNA-seq, also showed significant chromatin opening in at least one of the associated OCRs (Fig. 4F, 61 of 151 genes for cluster S4b). For the genes commonly exhibiting scRNA-seq and scATAC-seq increase, we find chromatin opening at a larger proportion of their associated regulatory regions compared to the genes that are not differentially expressed but show chromatin opening (Fig. 4G). We conclude that key pathogenesis driver genes are robustly activated only when cells simultaneously open at least a certain number of key regulatory regions associated with these genes. Overall, these results are consistent with the idea that chromatin remodeling of SFs is determinant in the formation of inflammatory arthritis.

**Fig. 5:**
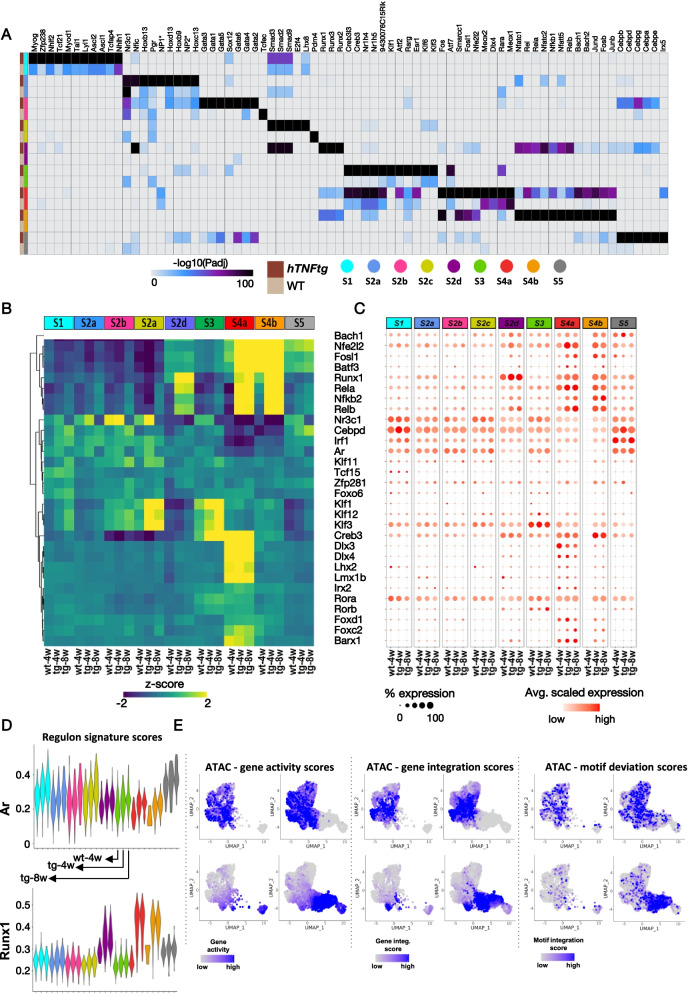
Integrative analysis of scATAC-seq and scRNA-seq infers putative arthritic regulatory programs. **A** Heatmap showing the motif enrichment *p*-adjusted values of each SF subpopulation, for each disease state (WT, *hTNFtg* as indicated). Motif enrichment analysis was performed within the disease-specific marker peaks depicted in Fig. 4D (right panel). The color signifies the magnitude of the enrichment (−log10 (*p*-adjusted value), hypergeometric test). **B** Heatmap showing the motif deviation *Z*-scores of positive TF regulators across the SF subpopulations and samples (WT: wt-4w; *hTNFtg*/4weeks: tg-4w; and *hTNFtg*/8 weeks: tg-8w). TF gene expression is positively correlated with motif TF accessibility (Pearson correlation > 0.5, *p*-adjusted value < 0.05). **C** Expression dot plot of positive TF regulators shown in **B**. The color of the dots shows the intensity of expression, and the size denotes the percentage of cells expressing the gene in each cluster and condition. **D** Violin plots of regulon gene signature scores across SF subpopulations and samples (wt, *hTNFtg*/4 & 8 as indicated). Top panel: gene signature of 23 Ar-regulated genes with significantly increased expression and chromatin accessibility in sublining clusters. Peaks are enriched with the Ar motif. Bottom panel: gene signature of 181 Runx1-regulated genes with significantly increased expression and chromatin accessibility in intermediate and lining clusters. Peaks are enriched with the Runx1 motif. **E** Multimodal feature plots of Ar and Runx1 including scATAC gene activity (ATAC—gene activity scores), scRNA expression embedded to scATAC cells (ATAC—gene integration scores), and scATAC TF motif activity (ATAC—motif deviation scores). *NP1 full name: NP_0322962_LINE6262_NP_0322962_I_N2. *NP2 full name: NP_0322962_LINE1459_NP_0322962_I_N31

Upon performing DNA motif analyses to determine which TF might control the cell-type or disease-specific regulatory regions and associated genes (Fig. 5A, Additional file 1: Fig. S9A), we highlighted cluster-specific groups of TFs dominating during disease: e.g., GATA family of TFs that regulate mesenchymal stem-cell differentiation transition (discussed in [60]) is linked to S2b (Osr1/Nr2f2+) while C/EBP family of TFs linked to S5(Ptx3/Notch3+) cluster, are involved in many processes including cell differentiation, inflammation, aging (discussed in [61, 62]). In contrast, S2d(Dkk3/Lrrc15+) and S4b(Birc5/Aqp1+) intermediate subpopulations are associated with Nfatc, which is known to play a central role in bone and joint remodeling during RA pathogenesis [63]. The S4a(Prg4^high^/Tspan15+) and S4b(Birc5/Aqp1+) clusters are linked to a combination of TFs including the chromatin remodeling mediators Smarcc1, Bach1/2, and the pro-inflammatory effectors Junb/d, Rel, and Nfkb (Fig. 5A, Additional file 1: Fig. S9B). TF binding sites (TFBS) that appear in diseased cells (within peaks found to be more accessible in *hTNFtg* SFs) revealed Rel, Nfkb, Junb/d, and Runx1 TFBS (Fig. 5A). We corroborated this finding by inferring the co-accessibility scores of regulatory regions modeled per-cell by employing cisTopic [64] (Additional file 1: Fig. S9C,D). We identified 12 topics that show distinct contribution probabilities along the SFs (Additional file 1: Fig. S9E, F). In particular, topic 12 matches S4a(Prg4^high^/Tspan15+) subpopulation, topic 5 matches S4b subpopulation, and topic 8 matches S4b(Birc5/Aqp1+) and S2d(Dkk3/Lrrc15+) subpopulation (Additional file 1: Fig. S9E, F). Motif analysis was applied on the regions defining these topics and confirmed that the intermediate and lining states are controlled by master regulators including Klf, Dlx, Creb3, Runx1, and Nfkb (Additional file 1: Fig. S9G).

We resolved true “positive TF regulators” by establishing which TFs show a high correlation between motif accessibility and TF mRNA expression at a single-cell resolution [39] (Fig. 5A, Additional file 1: Fig. S9A). The most deviant TFs were detected in the expanding intermediate and lining clusters (S2d(Dkk3/Lrrc15+), S4b(Birc5/Aqp1+), S4a(Prg4^high^/Tspan15+)) and to a lesser degree in the S5 subpopulation (Fig. 5B). While we noticed stable high deviation scores for a subset of TF regulating the Prg4^high^ lining cluster (Dlx, Lhx, and Lmx), we highlighted notable changes in TF regulatory programs (regulons) in disease for the intermediate and lining cells S4a(Prg4^high^/Tspan15+), S4b(Birc5/Aqp1+), and S2d(Dkk3/Lrrc15+) (compare healthy joint vs early and established disease states). These regulons are operated via the TFs Nfkb, Rela, Relb, Rel, and Runx1 (Fig. 5B, D and Additional file 1: Fig. S9B). Although the other Thy1+ sublining clusters show lower deviation scores and less dynamic changes, we noted that they are controlled via Klf, Cebpd, Ar, and Nr3c1 TFs. We validated these findings by verifying that the underlying expression scores of the TFs Ar and Runx1 as well as the genes they control (gene regulatory networks (GRNs)) parallel the motif deviation patterns (Fig. 5C, D, Additional file 1: Fig. S10A, B and Additional file 6: Table S5).

### A defined trajectory yields pathogenic SFs in diseased joints

**Fig. 6:**
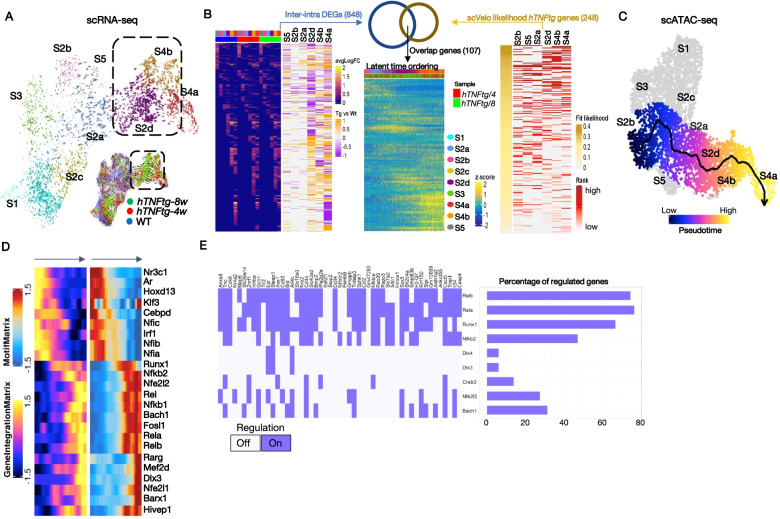
Inference of SF trajectories in the arthritic joint by RNA velocity analysis. **A** RNA velocity analysis recapitulating cell transitions and dynamic relations between SF clusters in the *hTNFtg* samples. Large panel: the UMAP highlights the existence of a pathogenic branch comprising S2d - S4b - S4a. Small panel: RNA velocity analysis in WT and *hTNFtg* samples. **B** Overlap of differentially expressed genes with scVelo driver genes, indicates genes potentially related to disease progression. In the first heatmap (left), avgLogFC values for DE genes, as calculated from inter-cluster and intra-cluster comparisons in each sample, are shown. In the second heatmap, binary values signify upregulation (orange) or downregulation (purple) of those genes in the *hTNFtg* vs WT comparison. In the third heatmap (right), genes are ranked according to the likelihood to drive the underlying cellular process. In the fourth heatmap (center), the scaled expression of the 107 overlapping genes is plotted. Cells are ordered by latent time values, after an S2b cell was set as the root of the trajectory. The gene expression patterns reveal a transcriptional gradient along the latent time axis in the *hTNFtg* SFs. **C** scATAC-seq semi-supervised trajectory analysis supports the existence of the aforementioned pathogenic branch. The color indicates the cellular fate across the inferred trajectory. **D** Heatmap showing the integrated gene expression activity (left panel) and the TF motif deviation (right panel) of positive TF regulators along the pseudotemporal ordered cells in the S2b- S2a - S2d - S4b - S4a branch. TFs gene expression is significantly correlated with TF motif deviation across the cell trajectory. **E** Binary heatmap of disease-related TFs and genes epigenetically primed for disease activation. Purple denotes that the TF regulates the gene, while white denotes a lack of regulation. The barplot summarizes the percentage of genes regulated by each TF

We next questioned which cells give rise to the emerging S2d, S4b, and S4a SF states in disease. We performed cellular trajectory analysis and traced the cells along an underlying Markov process to determine their respective latent time and identify plausible root cells. Root properties were mainly found in the S2b (Osr1/Nr2f2+) state cells albeit cells in S5(Ptx3/Notch3+), S4b(Birc5/Aqp1+), S1(Smoc2/Col15a1), and S3(Dpp4/Pi16+) SFs also exhibited a root-like potential (Fig. 6A, Additional file 1: Fig. S11A, B). Regardless of the origin, the cells transitioned via S2b, S2d, and S4b and always ended in the area of S4a(Prg4^high^/Tspan15+) (Fig. 6A, Additional file 1: Fig. S11B). Consistent with the TNF dependence of our murine arthritic model and the RNA velocity analysis outcome, we detected high activity scores for “response to TNF” S2b (Osr1/Nr2f2+) and S5(Ptx3/Notch3+) as well as for the expanded S2d, S4a, and S4b clusters (Additional file 1: Fig. S11E, upper panel). We further identified that a subset of S4b (Birc5/Aqp1+) cells adjacent to S4a(Prg4^high^/Tspan15+) showed activation of *Cdk1* and *Ccnb1* genes (Fig. 2D) and preferential expression of G2/M phase markers (Additional file 1: Fig. S11D) indicating that proliferation partially explains the increased abundance of the aforementioned cells in *hTNFtg* mice.

To understand potential functional relationships within the inferred cellular process, we reconstructed transcriptome dynamics considering the DE status and cell position in the proposed continuum. First, we focused on a subset of genes showing both cluster and disease specificity. The 848 genes isolated from 2,322 inter-cluster DE genes (Additional file 7: Table S6) were affected during the transition of cells into the intermediate (S2d and S4b) and pathological lining states (S4a) (Fig. 6B). A total of 107 of these genes, which were also identified by scVelo as drivers of the differentiation process thanks to their high likelihood gene scoring (Additional file 7: Table S6), were certified to play crucial roles in the progression of disease (GO analysis, Additional file 8: Table S7). Assignment of those genes in three main categories—early, intermediate, and late activation based on the output of hierarchical clustering of the gene expression scores—revealed the structure of the transcriptional pattern driving cellular changes from the initial to the final state, highlighted by genes such as *Runx1*, *Cd44*, *Tnfaip3*, and *Tnfaip*6, *Icam1*, or *Inhba* (Fig. 6B, Additional file 7: Table S6).

Pseudo-temporal ordering of the cells recapitulated at the epigenetic level the pathogenic transitions observed with scRNA-seq (trajectory inference from scATAC-seq datasets [37]) (Fig. 6C). We then sought to detect functional relationships and highlight regulators/TFs that drive the differentiation during pathogenicity. We analyzed the transcriptome dynamics considering the DE status and cell position in the proposed continuum and motif accessibility (see the “Methods” section). In accord with the regulon analysis, we found that Runx1 denotes a “switch” activating the expansion and development of disease-specific S2d(Dkk3/Lrrc15+), S4b(Birc5/Aqp1+), and S4a(Prg4^high^/Tspan15+) subpopulations and directly drives 27 of the 107 genes we defined as essential to arthritogenicity (the “Methods” section and Additional file 8: Table S7), while TFs like Rel, Nfkb2, and Dlx3 are key effectors of this process (Fig. 6D). Together, these results suggest that the expansion of the S2b-S2a-S2d-S4b-S4a branch upon TNF expression commands arthritis development and influences cell fate choices via specific sets of pathogenesis induced genes.

### Arthritogenic potential is epigenetically primed at the root of SF trajectory

We next assessed whether the choice in SF cells trajectory could be epigenetically primed for disease-promoting activity. We first examined which genes are transcriptionally inactive in the S2b SFs, the main root-cluster of our defined pathogenic lineage in both WT and *hTNFtg* samples. We focused on those transcripts that were activated at later cell states of the trajectory (S2a, S2d, S4b, S4a), and were also upregulated in *hTNFtg* SFs, compared to the naïve conditions. We then opted for genes that their promoters show a significant opening in the root state of the particular lineage (S2b (Osr1/Nr2f2+)), ending up to a cohort of 51 “primed” genes (Additional file 1: Fig. S12A, B). Most of these genes showed an enrichment of scATAC-seq signal in their linked distal regulatory elements (data not shown). These putative enhancers (peak-to-gene links) might be engaged in boosting the transcriptional activity of these genes when the arthritogenic TNF is present. Further exploration of the positive regulator analysis revealed that the previously identified disease-important TFs (Nfkb2, Rela, Relb, Runx1, Creb3, Nfe2l2, Bach1) preferentially target the regulatory regions of these genes (Fig. 6E, Additional file 1: Fig. S12C-enrichment analysis), indicating the high potential for these already opened sequences to initiate transcriptional circuits operating in disease initiation and progression. Notably, NFkB components substantially underlie the transcription of the most primed genes (Additional file 1: Fig. S12C). Functional enrichment further supported that the primed genes are heavily involved in inflammatory response, arthritis-promoting functions (*Ccl2*, *Cxcl5*, *Sphk1*) and, interestingly, Wnt pathway (*Bmp2*, *Rspo3*, *Cd44*) and stem cell differentiation (*Sox5*, *Nrp2*, *Cdk6*) (Additional file 1: Fig. S12D). Conclusively, our analytic approach assigns an epigenetic prospective in arthritogenesis, underlined by both the inflammatory activity and the plasticity of the specific SF subclusters.

### Common transcriptional modules control SFs in human RA and murine *hTNFtg* inflammatory arthritis

**Fig. 7:**
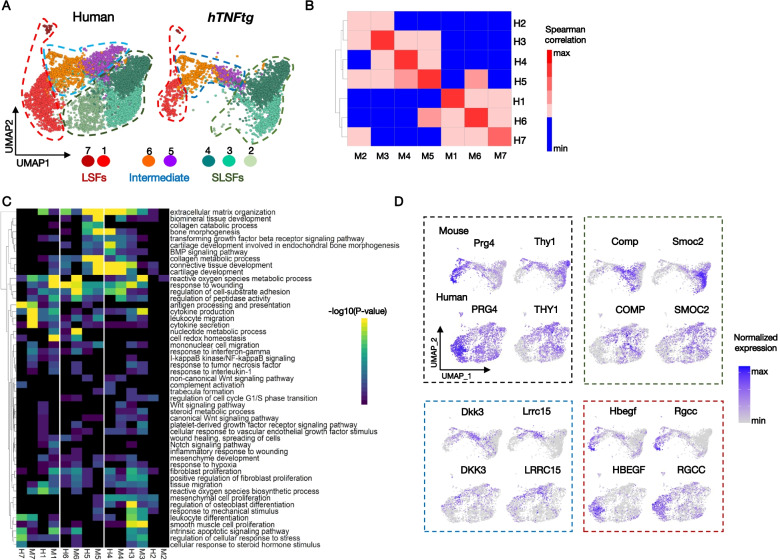
Integrative analysis of SFs from *hTNFtg* murine model and human RA pathology. **A** Integration of 24,042 RA patients’ SFs (3 different studies: Zhang et al. (2019), Wei et al. (2020), and Stephenson et al. (2018)) and our 3,051 *hTNFtg* SFs identified 7 SF clusters. UMAPs for the pooled human (downsampled to 3,051 cells) and mouse datasets, cells are colored by cluster identity. **B** Correlation heatmap (average expression of most variable genes) between human and mouse SF clusters. **C** Heatmap illustrating the significance of the selected enriched functional terms and pathways in human and mouse datasets. **D** Feature plots of selected marker genes commonly expressed between homologous human and mouse clusters of SF subpopulations

We integrated the previously generated scRNA-seq data from synovial biopsies of RA patients (H) [16, 17, 19], with our *hTNFtg* scRNA-seq dataset (M) (see the “Methods” section). We found that cells of both species align particularly well in the newly defined UMAP space. Unbiased graph-based clustering identified seven sub-populations (H1-H7; M1-M7) (Fig. 7A, Additional file 1: Fig. S13A-C). Correlation heatmap of the MVGs between human (H) and mouse (M) clusters revealed significant similarities in SF expression programs in the two species, albeit for cluster 2 that contains human SLSFs and only few mouse cells derived from the SLSFs that we described above (Fig. 7A). The mouse SLSF populations S1(Smoc2/Col15a1), S2a(Comp/Sfrp1+), S2b(Osr1/Nr2f2+), S2c(Meox1/Clu+), S3(Dpp4/Pi16+), and S5(Ptx3/Notch3+) located principally to clusters 3 and 4 and matched previously annotated human sublining cell expression profiles (Fig. 7B, Additional file 1: Fig. S13D). Cluster 1 and, to a lesser extent, cluster 7 brought together the human and murine lining Prg4^high^ cells (Fig. 7B, Additional file 1: Fig. S13A). They also contain a previously under-appreciated proliferative mixed lining/sublining SF state (see below), fitting with the idea of their cellular expansion in diseased joints. Cluster 5 contains the bulk of the mouse S2d(Dkk3/Lrrc15+) SLSFs and M5 is linked to human cells in both clusters 5 and 6, suggesting that both human clusters (H5 and 6) likely acquire the “intermediate” arthritis-specific profile previously identified in *hTNFtg* SF states (Fig. 7B, Additional file 1: Fig. S13A, D).

Functional inter-species similarities were confirmed via GO and pathway enrichment analyses of marker genes and co-clustering of (H) and (M) groups (Additional file 9: Table S8 and Additional file 10: Table S9). We highlight conserved functions and processes of SLSFs in regulating vasculogenesis, cell proliferation, muscle tissue development, bone and tissue renewal (clusters H3, M3, H4, and M4) (Fig. 7C). We demonstrate that M5 and H5 clusters are marked by pathogenic RA features such as metalloproteinase secretion, collagen catabolic processes, and bone destruction signaling pathways, further supporting the similarities with the S2d(Dkk3/Lrrc15+) SFs in *hTNFtg* model. Clusters 1, 6, and 7, which contain SFs from the lining synovial compartment that were previously acknowledged for their destructive properties, display pro-proliferative pathways but also appear to regulate immune-related and adhesion/migration pathways (Fig. 7C). In addition, key marker genes show reasonable levels of conservation between mouse and human data (Fig. 7D). As expected, the analysis of the more human-specific cluster 2 revealed less shared features, and significantly highlighted common functions associated with translation and ribosome assembly. The human H2 SFs further exhibit regulation of ossification, epithelial cell proliferation, and autophagy. On contrary, the gene expression of mouse M2 SFs points out functions associated with post-translational modifications and apoptotic cell death compared to H2 SFs (Additional file 9: Table S8, Additional file 10: Table S9).

**Fig. 8:**
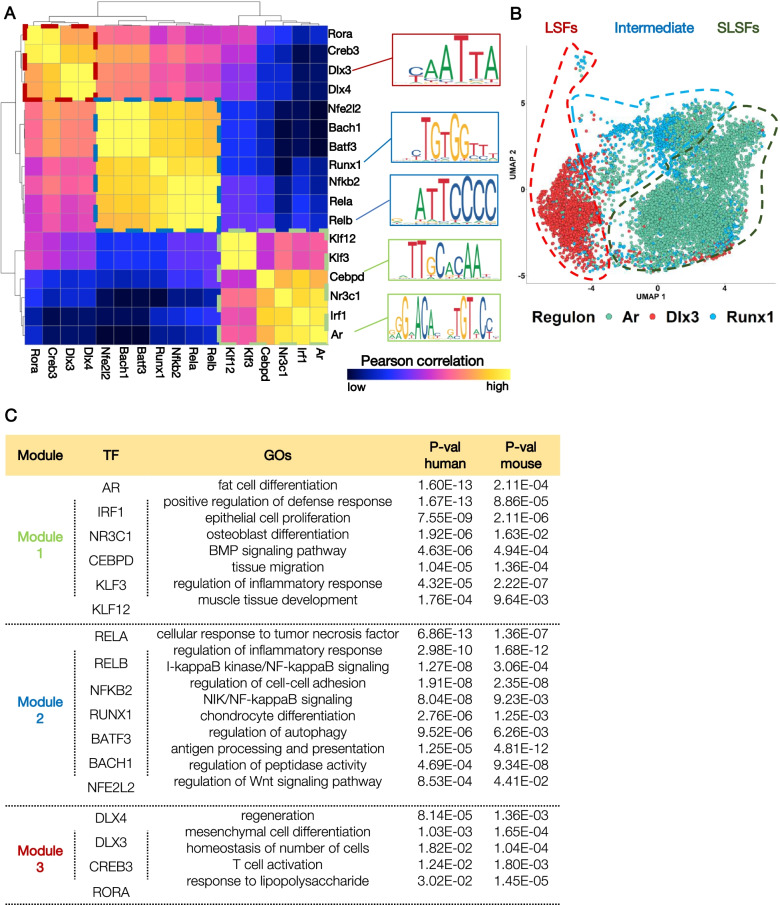
Shared Gene Regulatory Networks in SFs of *hTNFtg* mice and human RA. **A** Regulatory network analysis in mouse and human datasets reveals 17 shared regulons. Correlation and clustering analysis in the *hTNFtg* propose organization of those shared regulons in 3 main modules. **B** The activity of regulons AR, RUNX1, and DLX3 is depicted in a UMAP of the human data. **C** Summary table for the GO enrichment analysis in the target genes of the modules shown in **A**. For each module, the TFs can be found in the second column. In the third column, commonly enriched GOs for mouse and human regulons in each module are presented followed by their respective *p*-values

At the regulatory level, analysis of human and mouse data using the SCENIC algorithm [29] allowed the inference of common TF regulons across species. Briefly, we first identified co-expressed genes to formulate putative regulatory links and retained only those with a direct motif relationship between genes and TFs. Finally, we scored each regulon in each cell using AUC analysis (see the “Methods” section). We then preserved all the common and conserved TFs operating in datasets from the RA patients and arthritic mice. We identified the mouse regulatory modules (clusters of TFs) by applying pairwise correlation between the motif deviations of the mouse/human conserved TFs, and applied hierarchical clustering, as previously described [65]. This approach identified three main regulatory modules defining lining, intermediate, and sublining states and demonstrate a substantial overlap across species (Fig. 8A). Regulons are governed by Ar, Dlx3, and Runx1 TF activities (Fig. 8B) and GO enrichment analysis of TF and downstream genes (Fig. 8C) indicated the modules shared functionalities in both species: module one (Ar) controls multipotent functions of the main core of SLSFs; module two (Runx1) conducts functions reflecting a rather inflammatory profile, consistent with the intermediate profile of our *hTNFtg* SLSFs. Interestingly, we find up to 25 of the 107 core mouse genes as target genes in human cells (Additional file 11: Table S10), highlighting the translational potential for genes like *Tnfaip3* and *6*, *Tlr2*, *Lrrc15*, and *Bmp2*. Of note, module three (Dxl3) exhibits less acknowledged functions, which should be related to the lining SF profile of human and mouse SFs (Fig. 8C, Additional file 11: Table S10).

In conclusion, our integrative approach establishes shared mouse-human SF subsets with highly similar chromatin and transcriptional programs and functional characteristics.

## Discussion

In normal joints, the SFs facilitate joint maintenance by sustaining the quality of synovial membrane and synovial fluid. However, in RA, the synovium is progressively compromised due to unresolved inflammation, and this, ultimately, leads to loss of joint function. The mechanisms underlying the synovial homeostasis or sustenance of inflammation in RA still remain poorly defined. Previous studies originally segregated the SFs (lining and sublining) according to their transcriptional profiles and spatial distribution during acute murine arthritis, which corresponded well to respective RASF profiles. In this study, we created a blueprint of synovial fibroblast profiles in both homeostasis and TNF-mediated chronic arthritis by uncovering, at a single-cell level, their transcriptomic profiles, their spatial distribution, and the underlying regulatory networks that characterize the transition to TNF-mediated arthritic pathology.

We report here for the first time that the normal synovium exhibits different SF states which reflect the complexity of the SF tissue, serving different functions to maintain homeostasis. The Thy1− LSFs regulate lining layer size through apoptotic and migrative properties. According to their specific transcriptomic signature, LSFs directly respond to wounding, whereas the mechanisms to regulate mitochondrial calcium levels possibly contribute to the proper signaling alertness. Owing to their mesenchymal origin, normal SF sublining states segregate by their responses to growth factor and differentiation signals such as WNT, BMP, and TGFbeta. In line with the variety of elicited responses and the diversity of observed states, our GO analysis was essential to fully appreciate the related functionalities and the transcriptional regulators of SLSF (Thy1+) clusters regarding angiogenesis control (Pi16/Dpp4+ cluster (S3)), osteogenic processes (Comp/Sfrp1+ cluster (S2a)), chondrogenesis, and muscle development (Smoc2/Col15a1(S1)). By exhibiting decreased cellular proportions during the arthritic process, each SLSF subtype loses some homeostatic functions and acquires activated characteristics, indicating significantly complicated networks operating during arthritogenic process. Concomitantly with the shrinkage of the stably present SLSFs, we observed the emergence of arthritis-specific Thy1+ SF subpopulations (Dkk3/Lrrc15+ and Birc5/Aqp1+), accompanied by expansion of Prg4^high^/Thy1-LSFs. The Dkk3/Lrrc15+ arthritic SF profile (S2d) is defined by the two markers that had been individually described and recently linked to emerged pathological states of fibroblasts in RA [19] and other inflammatory and cancerous human conditions, respectively [44, 66]. The Birc5/Aqp1+ (S4b) expanded cluster additionally share a high Prg4 expression pattern and other features with lining Thy1− SFs (Prg4^high^/Tspan15+ (S4a)), and it is partly marked by Dkk3. All these emerged SFs share highly inflammatory and destructive properties, while the Birc5/Aqp1+(S4b) SFs are further characterized by high proliferative and DNA imprinting capacity, indicative of the structural and epigenetic changes reported for RA [67–69]. In line with this, a recent elegant study analyzing the inflammatory memory of SFs as a possible mechanism to explain flares in RA, identified arthritis-“primed” SLSFs (Thy1+) functioning in a mixed inflammatory/destructive mode upon arthritogenic restimulation [70]. Therefore, while previous studies highlighted the expansion of SLSFs and the distinct divergence of functions for Thy1+(SL) and Thy1-(L) SFs in arthritic disease, our comparative analysis indicates not only the structural and functional rearrangements but also the expansion of defined transcriptional SFs states commencing early in arthritic synovium and acting in dual inflammatory/destructive manner.

The observed expansion of specific SF states in arthritic mice could be suggestive of a TNF-mediated pattern of SF differentiation during disease development. This hypothesis is advocated by the lineage inference showing the major differentiation queue towards the emergence of these disease-specific clusters starts from the root fibroblast state Osr1/Nr2f2+ (S2b) SFs. The differentiation program always aims towards the Prg4^high^ SF state, indicating the fate of SLSFs (Thy1+) as a continuum towards LSFs (Thy1−) in disease. This transcriptional trajectory is in line with the expansion of the inflammatory lining profile (iS4a-Fig. 3) and indicates the Dkk3/Lrrc15+ (S2d) and Birc5/Aqp1+ (S4b) profiles as an intermediate stage in the progressive expansion and differentiation of the destructive SFs. The mouse/human integrative analysis identified that the DKK3/LRRC15+ SFs did exhibit significant expression similarities between species. In line with previous observations, they acquire an intermediate signature lying between the sublining/perivascular Notch3+ and the Prg4^high^ lining SFs in both human and murine (STIA) arthritic synovium (Additional file 1: Fig. S14, generated with publicly available datasets described in ref [20]). Interestingly, previous evidence for the origin and the emergence of common activated fibroblast states among tissues and human diseases including RA, suggested a different dominant root for the emergence of activated Lrrc15+ fibroblasts, that originate from Pi16+ or Col15a1+ fibroblasts [44]. In our system, the corresponding main clusters (Dpp4/Pi16+ (S3) and Smoc2/Col15a1+ (S1)) contribute less to the predicted roots of inferred trajectory. This likely indicates alternative activation pathways, which might be imprinted by the tissue and the specific arthritogenic signals (TNF) during *hTNFtg* disease. The heterogeneity of RA, the complex inflammatory cytokine network defining the cellular interactions, and the still-limited knowledge on whether and how the evolving SF states drive the pathogenicity and the destructive nature of arthritis, signifies the necessity for future targeted cell-fate mapping and functional studies.

The species-shared transcriptional modulators of the expansion of arthritic intermediate SFs are the NFkB pathway components NFkB1/2, RelA, and RelB, all well known as key regulators of inflammatory processes including inflammatory arthritic diseases [71, 72]. Notably, we had already addressed the SF-specific NFkB mediated responses in the development of TNF-mediated murine arthritis in a recent paper showing mechanistically how a major NFkB activator, the IKK2 kinase, acts as a dual modulator of arthritis through both the inflammatory and the death responses of SFs [6]. Owing to its robust upregulated expression in intermediate SF states, the Runx1 emerged in our analysis as another essential master regulator of DKK3/LRRC15+ SFs in both species. Besides hematopoiesis, Runx1 has been associated with osteochondral differentiation (along with Runx2 and 3) and fibroblast activation [73]. Consistently, Runx1 has been recently proposed as a dual inflammatory modulator and even an epigenetic modifier, depending on the context [74–80]. Runx1 has also been suggested as an important player in the context of RA and RASF pathogenicity in a study showing that an RA-associated SNP located in a super-enhancer, formed 3D contact with the promoter of RUNX1 gene in cytokine-stimulated RASFs. Authors further demonstrate that the knockdown of RUNX1 expression leads to the abrogation of the inflammatory output of stimulated RASFs and, therefore, revealed a crucial link of inflammatory gene expressions, epigenomic modulations in RUNX1 and RA susceptibility loci [81]. Our study strengthens this initial discovery and confirms the RUNX1 as a promising disease-paramount pathway that requires further studies.

By uncoupling the transcriptional cues with the unrealized epigenetic potential of the SFs, we also highlight genes such as *Sphk1* and *Pla2g2e*, the targeting of which had been previously shown to ameliorate modeled TNF-mediated arthritis [82, 83]. Similarly, the predicted ECM protein Tenascin C provides TLR-mediated amplification of inflammatory signaling in SFs and in murine models of RA [84]. Therefore, our analyses also dictate for previously underexplored gene targets in arthritis, such as *Rspo3* (effector molecule of WNT pathway) or *Ddit4* (hypoxia-induced, regulator of mTOR1 activity). In light of our and other lab results, additional studies are necessary to elucidate whether all the shared features among human RA and murine models and the predicted epigenetic potential depend solely on arthritogenic TNF signals and occur directly or indirectly, possibly through the secondary induction of Notch and/or other signaling pathways [70, 85].

## Conclusions

To date, this study is the first to compare the homeostatic and pathologic heterogeneity of SFs. Our analyses allowed to identify crucial sets of TFs and GRNs that cooperate to rewire SF identities and functions during the onset and progression of inflammatory arthritis. The alignment of our findings with the human context revealed a largely shared gene regulatory landscape that potentiate the added predictive value of our studies in prioritizing novel fibroblast-targeted diagnostic and druggable pathways for RA. Hence, our multiparametric data will serve as a key resource to the field for the formation and validation of additional novel mechanistic hypotheses on the pathogenic pathways operating in inflammatory arthritis.

## Supplementary Information

**Additional file 1: Figs. S1-S14**.

**Additional file 2: Table S1.** Cluster marker genes.

**Additional file 3: Table S2.** Functional enrichment analysis on mouse scRNA-seq DE gene lists.

**Additional file 4: Table S3.** Functional enrichment analysis S4.a sub-clusters.

**Additional file 5: Table S4.** Bulk RNA-seq Deseq2 files.

**Additional file 6: Table S5.** Regulated genes of Ar and Runx1.

**Additional file 7: Table S6.** Disease cluster specific markers, scVelo likelihood genes and 107 common genes.

**Additional file 8: Table S7.** Functional enrichment analysis on 107 disease driver genes.

**Additional file 9: Table S8.** Cluster marker genes, Human-Mouse analysis.

**Additional file 10: Table S9.** Functional enrichment analysis, Human-Mouse.

**Additional file 11: Table S10.** Target genes of interest and Functional enrichment analysis of Mouse-Human regulons.

## Data Availability

The raw and processed sequencing data reported in this study have been deposited with the BioProject under accession code PRJNA778928. All previously published datasets that were employed in the current study can be found in their respective public repositories, as described in the “Methods” section (mouse studies-STIA model: GSE129087 and human studies: GSE109450; phs001529.v1.p1; phs001457.v1.p1). The data analysis pipeline is available from the corresponding authors upon request.

## Abbreviations

DE: Differentially expressed
DEA: Differential expression analysis
GRN: Gene regulatory network
OCR: Open chromatin region
L SF: Lining synovial fibroblast
RA: Rheumatoid arthritis
sc: single cell
SF: Synovial fibroblast
SL SF: Sublining synovial fibroblast
TF: Transcription factor
TFBS: TF binding site
