# Supplementary material for: Single-cell multimodal analysis identifies common regulatory programs in synovial fibroblasts of rheumatoid arthritis patients and modeled TNF-driven arthritis
Source: Genome Med. 2022 Jul 26;14:78. doi: 10.1186/s13073-022-01081-3 (PMC9316748; doi:10.1186/s13073-022-01081-3)
Supplement: Supplementary file 1 — Additional file 1: Figs. S1-S14. [file 13073_2022_1081_MOESM1_ESM.pdf]

Additional file 1

Legends of Supplementary Figures

Fig. S1. scRNA-seq and scATAC-seq analysis of non-hematopoietic stromal PDPN+ cells derived from *hTNFtg* and WT ankles

(A). Representative hematoxylin and eosin staining of the ankle joint at the talus level from 4-week-old wt, 4- and 8-week-old *hTNFtg* mice. (B). Gating strategy for flow-cytometric analysis of synovial fibroblasts. Intact cells are identified based on forward scatter (FSC-A) and side scatter (SSC-A) characteristics. The dead cells (DAPI), leukocytes (CD45+), endothelial (CD31+) and pro-erythroblasts (Ter119+) were excluded from analysis. The cell sorting strategy for sc analysis is highlighted with the red gating. The sorting strategy for bulk RNA-seq is highlighted with the green gating. Pericytes that haven't been selected in any of the approaches are highlighted in the black doted (Pdpn-, Thy1+). (C). UMAP projection showing the different cell types in the pooled scRNA-seq dataset (left panel). Barplots showing the relative abundances of the different cell types (center panel). Dotplot showing scaled normalized expression of marker genes across the different cell types in the pooled RNA-seq dataset (right panel). (D). UMAP projection showing the different cell types in the pooled scATAC-seq dataset. Barplots showing the relative abundances of the different cell types in the pooled scATAC-seq dataset. (E). Barplots depicting the number of genes detected per cell (upper panel, left) and the number of total reads per cell (upper panel, right) in the pooled RNA-seq dataset including only fibroblast cells. At the bottom left panel sample-specific TSS enrichment profiles (WT, *hTNFtg*-w4 and *hTNFtg*-w8 weeks as indicated) showing a sharp peak at TSS and a smaller peak (right of TSS) due to the stably positioned +1 nucleosome. At the right panel, sample-specific fragment size distributions showing negligible variability across samples (wt, *hTNFtg*-4w and *hTNFtg*-8w weeks as indicated). (F). Barplots showing the relative abundances of fibroblast clusters in scRNA-seq (left) and scATAC-seq datasets (right). (G). Dotplot showing scaled normalized expression of fibroblast markers for the different clusters in the pooled RNA-seq dataset containing only fibroblasts.

Fig. S2. Differential Expression Analysis (DEA) of *hTNFtg* and WT SF subtype transcriptomes (relative to Figure 2)

(A). Barplots showing the percentage of Thy1 positive, Prg4 positive and double positive SF cells in each cluster per sample for scRNA-seq (top) and scATAC-seq (bottom) modalities. (B). SF clusters correlation heatmap of most variable genes in the pooled dataset (*wt*, *hTNFtg*-4w and *hTNFtg*-8w). Individual clusters and samples are color-coded. Pathology and homeostasis specific highly correlated group of clusters are highlighted with black dotted lines. (C). Table summarizing the number of genes commonly up-regulated in inter- and intra- (*hTNFtg* vs WT) cluster DEA for selected clusters and (bottom) circos plot visualizing the extent of shared up-regulated genes between clusters S2d, S4b and S4a. (D). Functional enrichment analysis of intra-DEGs (*hTNFtg* vs WT) for the clusters S2d, S4b andS4a. Functional terms in the heatmap are colored according to statistical significance of the enrichment.

Fig. S3. Unique functions of WT SFs (relative to Figure 2)

Functional enrichment analysis of positive cluster marker genes (AvgLogFC >= 0.25 & Pval < 0.01) for identification of unique functions evident only in WT SF subtypes. The color of the dot signifies the statistical significance of the enrichment, while the size of the dot denotes the number of marker genes of the cluster included in the term.

Fig. S4. Unique functions of *hTNFtg* SFs (relative to Figure 2)
(A). Functional enrichment analysis of positive cluster marker genes (AvgLogFC >= 0.25 & Pval < 0.01) for identification of unique functions evident only in *hTNFtg*-w4 SF subtypes. (B). Functional enrichment analysis of positive cluster marker genes (AvgLogFC >= 0.25 & Pval < 0.01) for identification of unique functions evident only in *hTNFtg*-w8 SF subtypes. The color of the dot signifies the statistical significance of the enrichment, while the size of the dot denotes the number of marker genes of the cluster included in the term.

Fig. S5. Gain and loss of functions in SF subtypes during disease (relative to Figure 2)
(A). Dotplot summarizing the gained enriched functional terms in *hTNFtg* SF subtypes compared to WT (top70 terms are shown, selected by Padj). (B). Dotplot summarizing the lost enriched functional terms at established state of disease (*hTNFtg*-w8) compared to healthy (WT) and early disease (*hTNFtg*-w4) states.
The color of the dot signifies the statistical significance of the enrichment, while the size of the dot denotes the number of marker genes of the cluster included in the term.

Fig. S6. Integrative analysis of SFs from healthy mice, *hTNFtg* and STIA murine models of Arthritis
(A). Projection of SFs in UMAP space after integration of the three samples (WT, *hTNFtg*, STIA). Cells are colored by cluster identity (clusters were identified during integration analysis). (B). Barplot showing relative abundances of the integrated clusters in the three samples (WT, *hTNFtg*, STIA). Clusters 1, 2, 5 and 6 are increased in both arthritic samples compared to WT. (C). Projection of SFs in UMAP space after integration of the three samples (WT, *hTNFtg*, STIA). Cells arecolored by original clustering annotation (before identification of integrated clusters). (D). Barplot showing relative abundances of the original cluster annotations in the three samples (WT, *hTNFtg*, STIA). (E). Dotplot depicting similarities in gene expression patterns of sublining (highlighted area in the bottom), intermediate (highlighted area in the middle) and lining (highlighted area in the top) marker genes between *hTNFtg* and STIA samples. The color of the dot signifies the scaled average expression value, and the size denotes the percentage of expression per cluster. (F). Correlation heatmap of scaled expression values for most variable genes (identified during integration) indicates similarities between clusters of *hTNFtg* and STIA samples.

Fig. S7. Spatial distribution of the SFs clusters in the WT and *hTNFtg* ankle joints (relative to Figure 2 and 3)
Representative confocal section (n=3 mice per genotype) depicting expression of marker genes identified upon sc transcriptomic analysis. Dashed yellow line in all the wt and *hTNFtg* images indicates, sub-lining localization, joint space (js), bone (b) and cartilage (c) are also labeled in each image. In the *hTNFtg* images dashed line highlights the destructive pannus that has invaded in the talus bone of the ankle joint. The marker genes and the associated subpopulations are indicated on each panel. (A). Expression of S2c marker Clu (purple) is distributed within the sublining compartment, distinctly from Prg4-expressing

cells (LSFs) in WT synovium. Gdf10(green) (S2a and b marker) colocalizes with Thy1+(red) SFs. Sema3C(red) (S3 marker) shows a dispersed expression in sublining synovium in WT joints (Absent from Prg4+(red) SFs), however the expression is further distributed in diseased sublining synovium (see the colocalization of Sema2c(green) with Thy1+(green) SFs). Notch3(green) S5 marker displays a perivascular localization in the healthy synovial membrane, co-localized within the Thy1+(purple) SFs, as also indicated with CD31(red) blood vessel specific so-staining, in both WT and diseased synovia. (B). Myofibroblast marker gene a-Sma(green) is not detected in the healthy synovium and it exhibits exclusive expression by pericytes. CD44 (green) and Comp(white) are co-expressed in the Thy1+ SFs of the healthy joint. In the *hTNFtg* joints, a-Sma (green) colocalizes with Thy1+(purple) and Thy1- SFs. CD44(green)/Ki67(white)+ SFs (marker combination for S2d and S4b clusters) are detected at the interface of pannus/ bone. Runx1 and Dkk3 (red) both display similar localization in both lining and sublining compartments, mainly at their intersection (indicated with arrows) (C). Prg4, main marker for the S4a lining SFs displays exclusive expression in the outermost SF layer of the healthy joint. In the *hTNFtg* joint, Prg4 expression is expanded and is mainly distributed in the interface of pannus/bone and sparsely within the rest of Thy1- synovium (indicated with arrows). All Scale bars, 50μm., b: bone, c: cartilage, js: joint space, l: lining and sl: sublining.

Fig. S8. Bulk RNA-sequencing analysis of WT, *hTNFtg*-w4 and *hTNFtg*-w8 sublining and lining samples (relative to Figure 2 and 3)

(A). PCA analysis of bulk RNA-seq samples shows that they are grouped horizontally as WT(left) and *hTNFtg*(right) and vertically as sub-lining(top) and lining(down). (B). Volcano plots showing up and down regulated genes for the comparisons Wt L vs Wt SL, *hTNFtg*-w4 L vs *hTNFtg*-w4 SL and *hTNFtg*-w8 L vs *hTNFtg*-w8 SL. (C). Scatter plot showing the extent of the overlap (and the correlation in Fold Changes) for lining, intermediate and sublining specific DEGs between scRNA-seq and bulk RNAseq. These genes are referred to as “co differentially expressed sc-bulk”. Note the lower numbers in *hTNFtg* samples. (D). On the left side, box plots showing Log2FC values (L vs SL comparisons) in each sample of bulk RNA-seq data for the positive marker genes of each cluster in the scRNA-seq dataset. On the right-side, error-bar plots showing the standard error of L2FC values for 3 selected marker genes per cluster. (E). Proportions of the marker genes found by scRNA-seq for a specific cluster, which are also up regulated in bulk RNAseq for lining (shades of red) and/or sublining cells (shades of blue). (F). Heatmap representing scaled normalized counts detected with bulk RNAseq assay for the genes co differentially expressed in sc and bulk described in panel (C). Hierarchical clustering by row highlights distinct lining, intermediate and sublining patterns (see Additional file 5: Table S4). (G). (left) Heatmap representing the fold changes (L vs SL) calculated in bulk RNA-seq assay, organized by sample specificities and only for the genes showing significant FC values ($|L2FC|>0.58$ and $Pval<0.05$) exclusively either in WT, or both *hTNFtg* samples, or *hTNFtg*-4w (Tg4) or *hTNFtg*-8w (Tg8) (Right). Aligned Heatmap representing scaled normalized counts detected with bulk RNA-seq assay. The red dashed boxes highlight candidate genes that can be used for creating a RT-PCR panel to test disease status by comparing Lining vs Sublining gene expression levels (see Additional file 5: Table S4).

Fig. S9. **Motif enrichment analysis in scATAC-seq accessible regions and cis-regulatory modeling on scATAC-seq data define distinct types of regulatory programs across SF subpopulations (relative to Figure 5)**
(A). Heatmap showing the motif enrichment P-adjusted values of each SF subpopulation. Motif enrichment analysis was performed within the SF marker peaks depicted in figure 1E (lower panel). Color signifies the magnitude of the enrichment (−log10 (P adjusted value), hypergeometric test). Columns are order by using binary sorting. (B). Feature plots of selected TF motifs with regulatory activity in the sublining, intermediate and lining SF subpopulations. Color signifies the motif deviation scores. (C). UMAP representation of SFs across the two disease states using cell-topic probabilities. (Wt and *hTNFtg* as indicated). Cells are colored by cluster identities and the marked area highlights the structural dynamic changes of the intermediate and lining subpopulations during disease progression. (D). CisTopic modelling of cis-regulatory topics using Latent Dirichlet Allocation. An optimal model of 12 topics was selected based on log likelihood. (E). Feature Plots of per-cell topic probabilities for representative modeled topics on the aggregated scATAC-seq dataset. (F). Heatmaps showing the per-cell topic z-scores (columns) for each modeled topic (rows), for each state (left panel: WT, right panel: *hTNFtg*). (G). Selection of representative motif enrichment results of the most contributing regions of topic 5, topic 8 and topic 12.

Fig. S10. Runx1 motif-enriched regulatory elements control the expression of Runx1 and Cd44 genes during the arthritic disease state (relative to Figure 5)
(A). (On the left panel) Genome track snapshot of the extended Runx1 gene locus (chr16, 92,576,073–92,926,074). (On the right panel) Single-cell gene expression (Log2 GeneIntegrationMatrix: Runx1) across SF subpopulations and disease states (wt, *hTNFtg*(Tg)) is shown. Merged peaks across SF subpopulations (Peaks), and differentially accessible peaks between SF subpopulations (All_DARs) are shown below. Differential accessible peaks with significantly increased scATAC-seq signal in S2d *hTNFtg* (S2d_DARs), S4b *hTNFtg* (S4b_DARs), and S4a *hTNFtg* (S4a_DARs) cells are also reported accordingly. Inferred peak-to-gene linkages for intragenic and distal intergenic regulatory elements are shown below (Peak2GeneLinks). Color scale signifies the level of correlation between peak scATAC-seq accessibility and integrated gene scRNA-seq expression (value). (B). (On the left panel) Genome track snapshot of the extended Cd44 gene promoter (chr2, 102,851,664–102,951,665). (On the right panel) Single-cell gene expression (Log2 GeneIntegrationMatrix: Cd44) across SF subpopulations and disease states (wt, *hTNFtg*) is shown. Merged peaks across SF subpopulations (Peaks), and differentially accessible peaks between SF subpopulations (All_DARs) are shown below. Differential accessible peaks with significantly increased scATAC-seq signal in S2d *hTNFtg* (S2d_DARs), S4b *hTNFtg* (S4b_DARs), and S4a *hTNFtg* (S4a_DARs) cells are also reported accordingly. Inferred peak-to-gene linkages for intragenic and distal intergenic regulatory elements are shown below (Peak2GeneLinks). Color scale signifies the level of correlation between peak scATAC-seq accessibility and integrated gene scRNA-seq expression (value).

Fig. S11. Ordering of the cells across the identified trajectory using latent time values and alternative methodologies (Relative to figure 6)
(A). RNA velocity recapitulating cell transitions and dynamic relations between SF clusters in the WT and the *hTNFtg* datasets (WT = blue, *hTNFtg*-w4 = red, *hTNFtg*-w8 = green). (B).

UMAPs of *hTNFtg* samples, cells are colored by their potential of belonging to the initial(left) or final state(right) of the identified trajectory (upper panel) and UMAPs of *hTNFtg* samples, cells are colored by latent time value (lower panel). In the latent time panels, the calculation performed by considering the root cell from clusters S2b, S5 and S3. (C). Partitions identified by PAGA algorithm and minimum spanning tree produced by Slingshot propose a similar global structure of the mouse data, supporting the existence of a trajectory backbone which includes clusters S2a, S2d, S4b and S4a. (D). Barplots showing the percentages of cells in each cluster (per sample) belonging to phase G1 (green), S (blue) and G2/M (orange). Cells shown in the UMAP (right panel) are colored according to the same color scheme. (E). UMAP projections of WT(left) and *hTNFtg*(right) samples, where cells are colored based on signature scores for the GO term "Response to TNF" (left panel) or on the normalized expression of Notch3 (right panel).

Fig. S12. Integrative analysis of scATAC-seq and scRNA-seq suggests 51 genes epigenetically primed for disease activation. (relative to Figure 6)
(A). Log transformed activity scores (scATAC-seq data) for the 51 genes are shown for the clusters of the pathogenic lineage in WT and *hTNFtg* conditions. (B). Scaled normalized expression values (scRNA-seq data) for the 51 genes are shown for the clusters of the pathogenic lineage in WT and *hTNFtg* (Tg) conditions. (C). Overlap of positive regulators from deviation motif analysis (Fig. 5B) and regulatory features predicted by TF enrichment analysis (i-cisTarget) highlights the TFs Rela, Relb, Nfkb2. (D). Functional enrichment analysis of the identified 51 genes. Selected enriched GO terms are presented in the barplot. Color gradient signifies the statistical significance of enrichment.

Fig. S13. Human/Mouse integrative analysis employing the available scRNA datasets from SFs of RA patients and the *hTNFtg* mice (relative to figure 7)
(A). UMAP projections showing the distribution of cells in the integrated clusters for the datasets used in human mouse integration analysis. (B). Stacked barchart showing the relative abundances of cells belonging to each of the integrated clusters in the datasets described in (A). (C). Cells from the 4 datasets used in integration analysis are plotted in the common integrated UMAP space and are colored by their original clustering annotation. (D). Stacked barcharts showing the distribution of cells (using the original clustering annotation) to the integrated clusters for the datasets shown in (C).

Fig. S14. Dkk3 and Lrrc15 expression in the intermediate transcriptional state of SFs in murine and human arthritis
(A). Feature plots of mouse synovial fibroblasts from Wei et al., 2020. Cells are colored by normalized expression of genes Thy1, Prg4, Lrrc15 and Dkk3. (B). UMAP projection(left) of mouse synovial fibroblasts shown in (a). Cells are colored by signature score. The signature score is calculated as the sum of scaled normalized expression values for the 71 intermediate genes described in Wei et al., 2020. Density plot(right) of the same signature scores for human RA synovial fibroblasts from Zhang et al., 2019.

Fig. S1

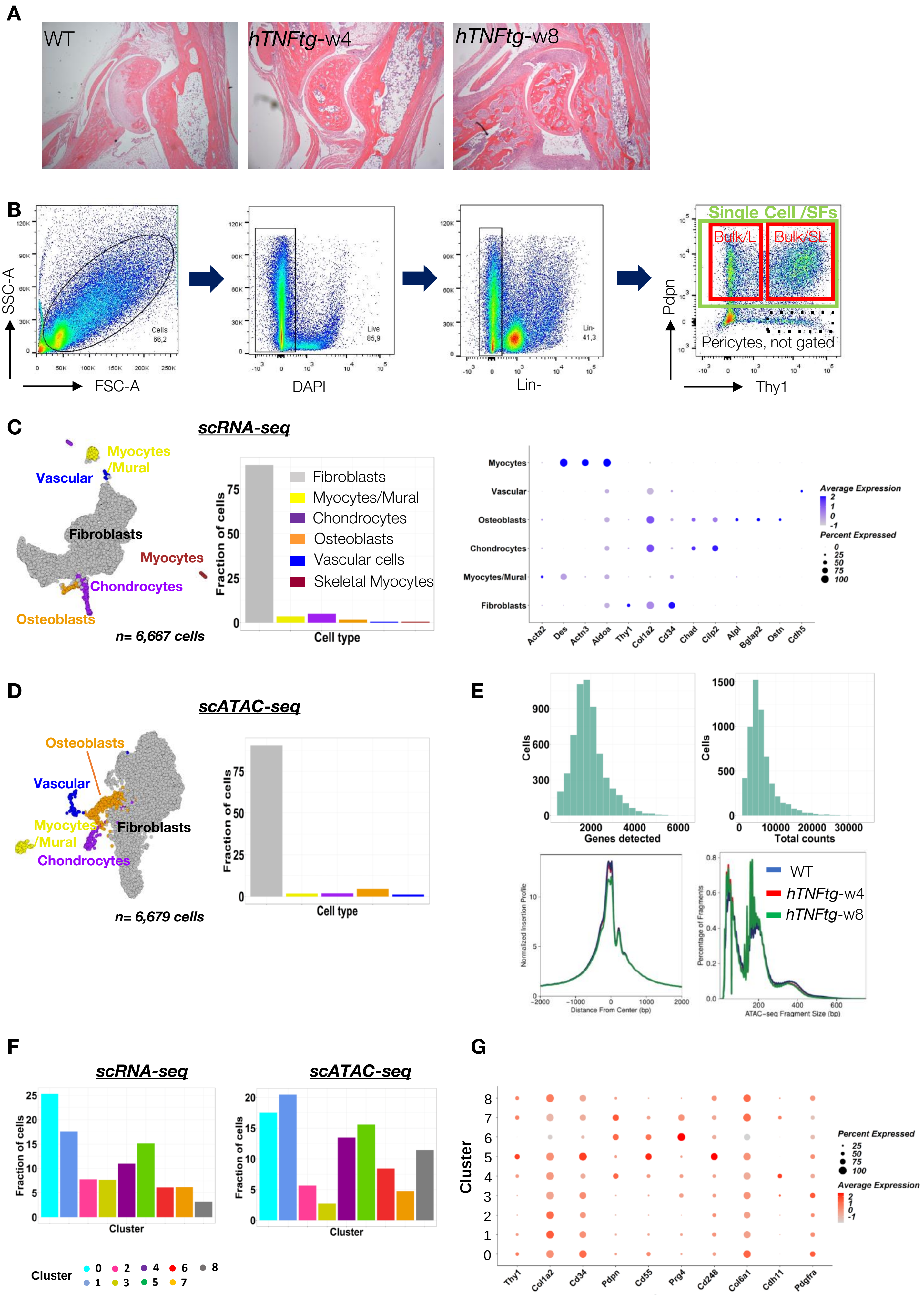
A
WT
hTNFtg-w4
hTNFtg-w8
B
SSC-A
FSC-A
Cells 66,2
DAPI
Live 85,9
Lin-
Lin- 41,3
Single Cell /SFs
Bulk/L
Bulk/SL
Pericytes, not gated
Pdpn
Thy1
C
scRNA-seq
Myocytes /Mural
Vascular
Fibroblasts
Myocytes
Chondrocytes
Osteoblasts
n= 6,667 cells
Fraction of cells
Cell type
Fibroblasts
Myocytes/Mural
Chondrocytes
Osteoblasts
Vascular cells
Skeletal Myocytes
Myocytes
Vascular
Osteoblasts
Chondrocytes
Myocytes/Mural
Fibroblasts
Acta2
Des
Actn3
Aldoa
Thy1
Col1a2
Cd34
Chad
Cilp2
Alpl
Bglap2
Ostn
Cdh5
Average Expression
Percent Expressed
D
scATAC-seq
Osteoblasts
Vascular
Myocytes /Mural
Fibroblasts
Chondrocytes
n= 6,679 cells
Fraction of cells
Cell type
E
Cells
Genes detected
Cells
Total counts
Normalized Insertion Profile
Distance From Center (bp)
Percentage of Fragments
ATAC-seq Fragment Size (bp)
WT
hTNFtg-w4
hTNFtg-w8
F
scRNA-seq
scATAC-seq
Fraction of cells
Cluster
Fraction of cells
Cluster
Cluster
G
Cluster
Thy1
Col1a2
Cd34
Pdpn
Cd55
Prg4
Cd248
Col6a1
Cdh11
Pdgfra
Percent Expressed
Average Expression

Fig. S2

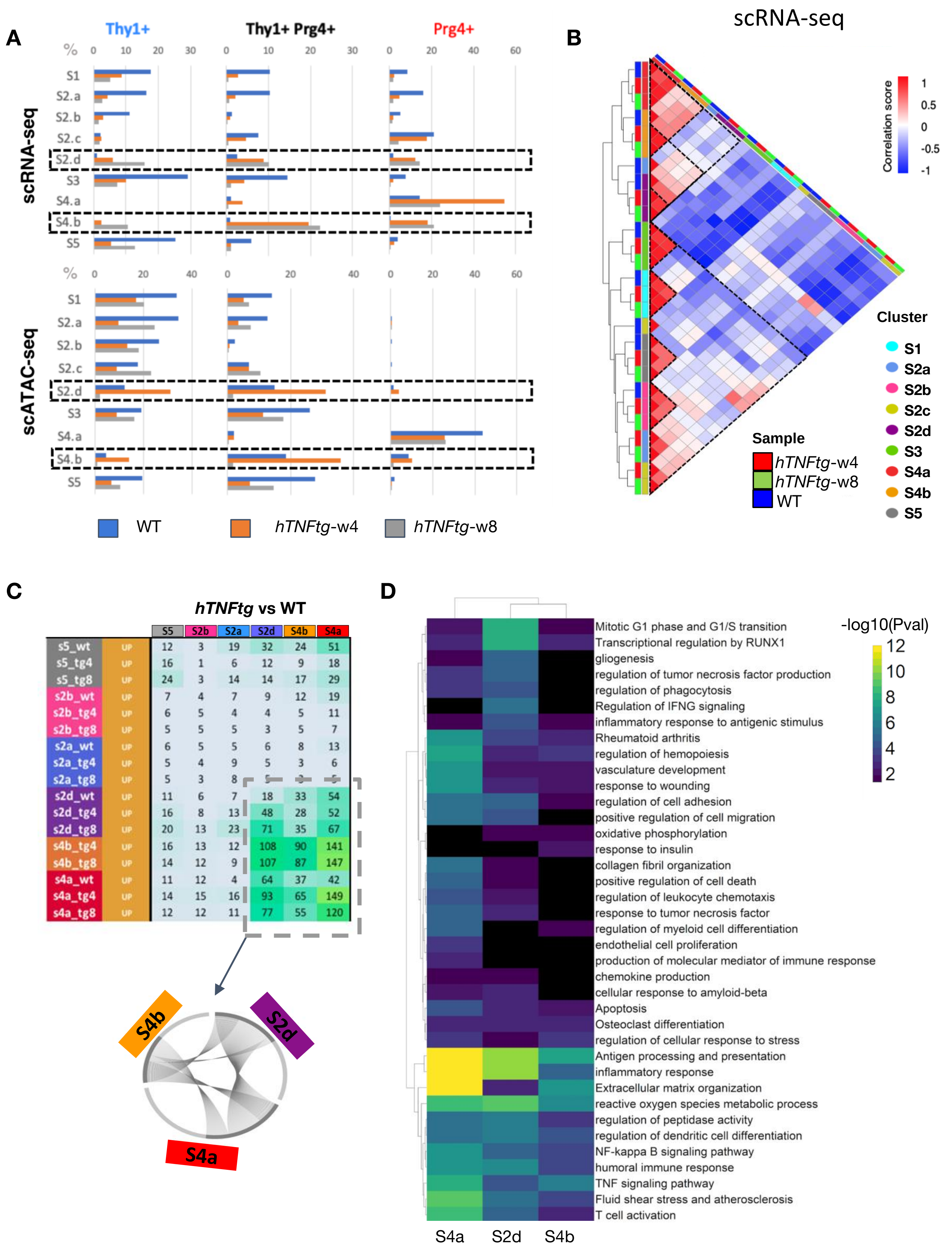
A
Thy1+
Thy1+ Prg4+
Prg4+
scRNA-seq
scATAC-seq
%
S1
S2.a
S2.b
S2.c
S2.d
S3
S4.a
S4.b
S5
WT
hTNFtg-w4
hTNFtg-w8
B
scRNA-seq
Correlation score
Sample
hTNFtg-w4
hTNFtg-w8
WT
Cluster
S1
S2a
S2b
S2c
S2d
S3
S4a
S4b
S5
C
hTNFtg vs WT
S5 S2b S2a S2d S4b S4a
s5_wt UP 12 3 19 32 24 51
s5_tg4 UP 16 1 6 12 9 18
s5_tg8 UP 24 3 14 14 17 29
s2b_wt UP 7 4 7 9 12 19
s2b_tg4 UP 6 5 4 4 5 11
s2b_tg8 UP 5 5 5 3 5 7
s2a_wt UP 6 5 5 6 8 13
s2a_tg4 UP 5 4 9 5 3 6
s2a_tg8 UP 5 3 8
s2d_wt UP 11 6 7 18 33 54
s2d_tg4 UP 16 8 13 48 28 52
s2d_tg8 UP 20 13 23 71 35 67
s4b_tg4 UP 16 13 12 108 90 141
s4b_tg8 UP 14 12 9 107 87 147
s4a_wt UP 11 12 4 64 37 42
s4a_tg4 UP 14 15 16 93 65 149
s4a_tg8 UP 12 12 11 77 55 120
S4b
S2d
S4a
D
-log10(Pval)
Mitotic G1 phase and G1/S transition
Transcriptional regulation by RUNX1
gliogenesis
regulation of tumor necrosis factor production
regulation of phagocytosis
Regulation of IFNG signaling
inflammatory response to antigenic stimulus
Rheumatoid arthritis
regulation of hemopoiesis
vasculature development
response to wounding
regulation of cell adhesion
positive regulation of cell migration
oxidative phosphorylation
response to insulin
collagen fibril organization
positive regulation of cell death
regulation of leukocyte chemotaxis
response to tumor necrosis factor
regulation of myeloid cell differentiation
endothelial cell proliferation
production of molecular mediator of immune response
chemokine production
cellular response to amyloid-beta
Apoptosis
Osteoclast differentiation
regulation of cellular response to stress
Antigen processing and presentation
inflammatory response
Extracellular matrix organization
reactive oxygen species metabolic process
regulation of peptidase activity
regulation of dendritic cell differentiation
NF-kappa B signaling pathway
humoral immune response
TNF signaling pathway
Fluid shear stress and atherosclerosis
T cell activation
S4a S2d S4b

Fig. S3

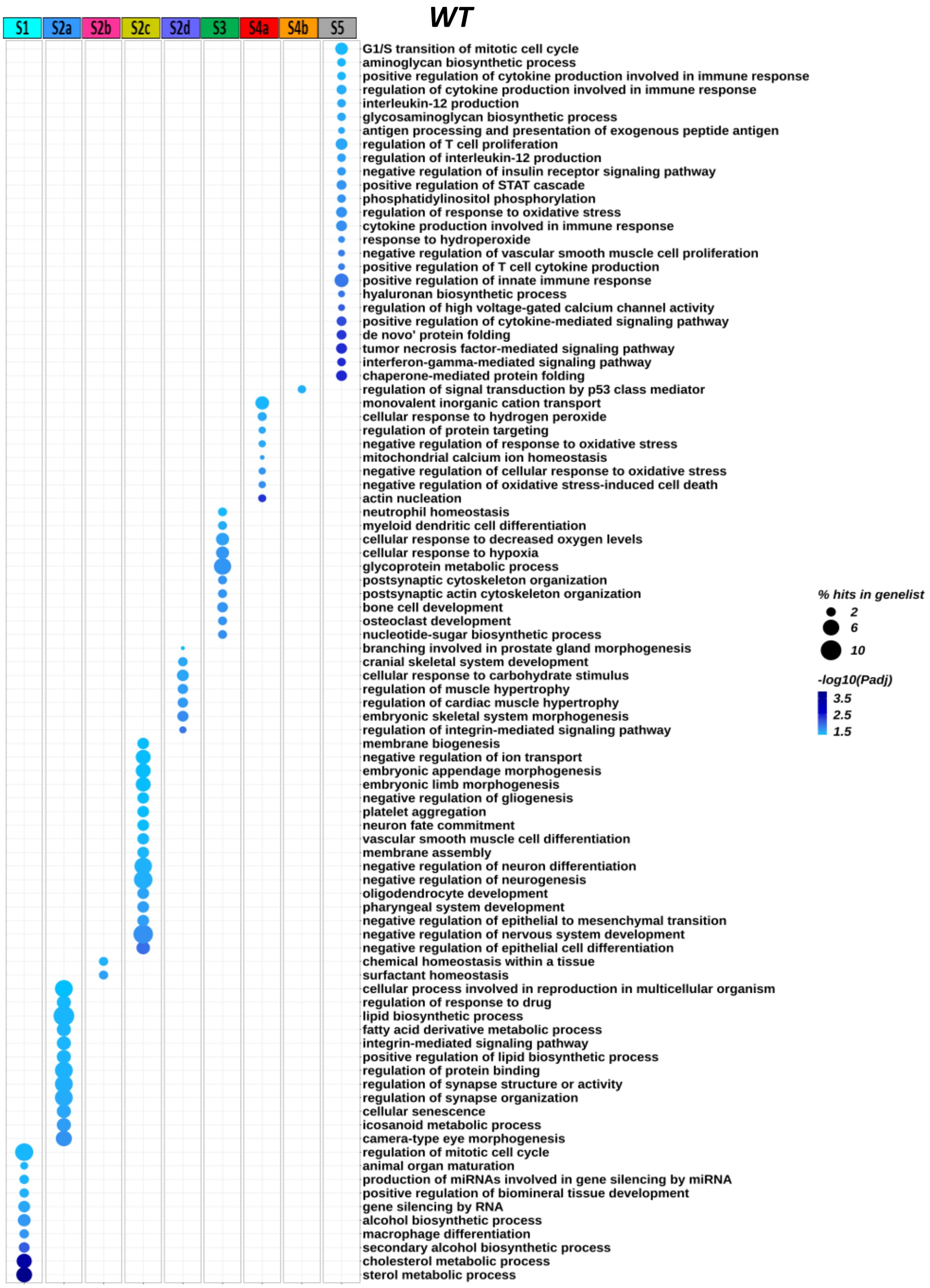
WT
S1
S2a
S2b
S2c
S2d
S3
S4a
S4b
S5
G1/S transition of mitotic cell cycle
aminoglycan biosynthetic process
positive regulation of cytokine production involved in immune response
regulation of cytokine production involved in immune response
interleukin-12 production
glycosaminoglycan biosynthetic process
antigen processing and presentation of exogenous peptide antigen
regulation of T cell proliferation
regulation of interleukin-12 production
negative regulation of insulin receptor signaling pathway
positive regulation of STAT cascade
phosphatidylinositol phosphorylation
regulation of response to oxidative stress
cytokine production involved in immune response
response to hydroperoxide
negative regulation of vascular smooth muscle cell proliferation
positive regulation of T cell cytokine production
positive regulation of innate immune response
hyaluronan biosynthetic process
regulation of high voltage-gated calcium channel activity
positive regulation of cytokine-mediated signaling pathway
de novo' protein folding
tumor necrosis factor-mediated signaling pathway
interferon-gamma-mediated signaling pathway
chaperone-mediated protein folding
regulation of signal transduction by p53 class mediator
monovalent inorganic cation transport
cellular response to hydrogen peroxide
regulation of protein targeting
negative regulation of response to oxidative stress
mitochondrial calcium ion homeostasis
negative regulation of cellular response to oxidative stress
negative regulation of oxidative stress-induced cell death
actin nucleation
neutrophil homeostasis
myeloid dendritic cell differentiation
cellular response to decreased oxygen levels
cellular response to hypoxia
glycoprotein metabolic process
postsynaptic cytoskeleton organization
postsynaptic actin cytoskeleton organization
bone cell development
osteoclast development
nucleotide-sugar biosynthetic process
branching involved in prostate gland morphogenesis
cranial skeletal system development
cellular response to carbohydrate stimulus
regulation of muscle hypertrophy
regulation of cardiac muscle hypertrophy
embryonic skeletal system morphogenesis
regulation of integrin-mediated signaling pathway
membrane biogenesis
negative regulation of ion transport
embryonic appendage morphogenesis
embryonic limb morphogenesis
negative regulation of gliogenesis
platelet aggregation
neuron fate commitment
vascular smooth muscle cell differentiation
membrane assembly
negative regulation of neuron differentiation
negative regulation of neurogenesis
oligodendrocyte development
pharyngeal system development
negative regulation of epithelial to mesenchymal transition
negative regulation of nervous system development
negative regulation of epithelial cell differentiation
chemical homeostasis within a tissue
surfactant homeostasis
cellular process involved in reproduction in multicellular organism
regulation of response to drug
lipid biosynthetic process
fatty acid derivative metabolic process
integrin-mediated signaling pathway
positive regulation of lipid biosynthetic process
regulation of protein binding
regulation of synapse structure or activity
regulation of synapse organization
cellular senescence
icosanoid metabolic process
camera-type eye morphogenesis
regulation of mitotic cell cycle
animal organ maturation
production of miRNAs involved in gene silencing by miRNA
positive regulation of biomineral tissue development
gene silencing by RNA
alcohol biosynthetic process
macrophage differentiation
secondary alcohol biosynthetic process
cholesterol metabolic process
sterol metabolic process
% hits in genelist
2
6
10
-log10(Padj)
3.5
2.5
1.5

Fig. S4

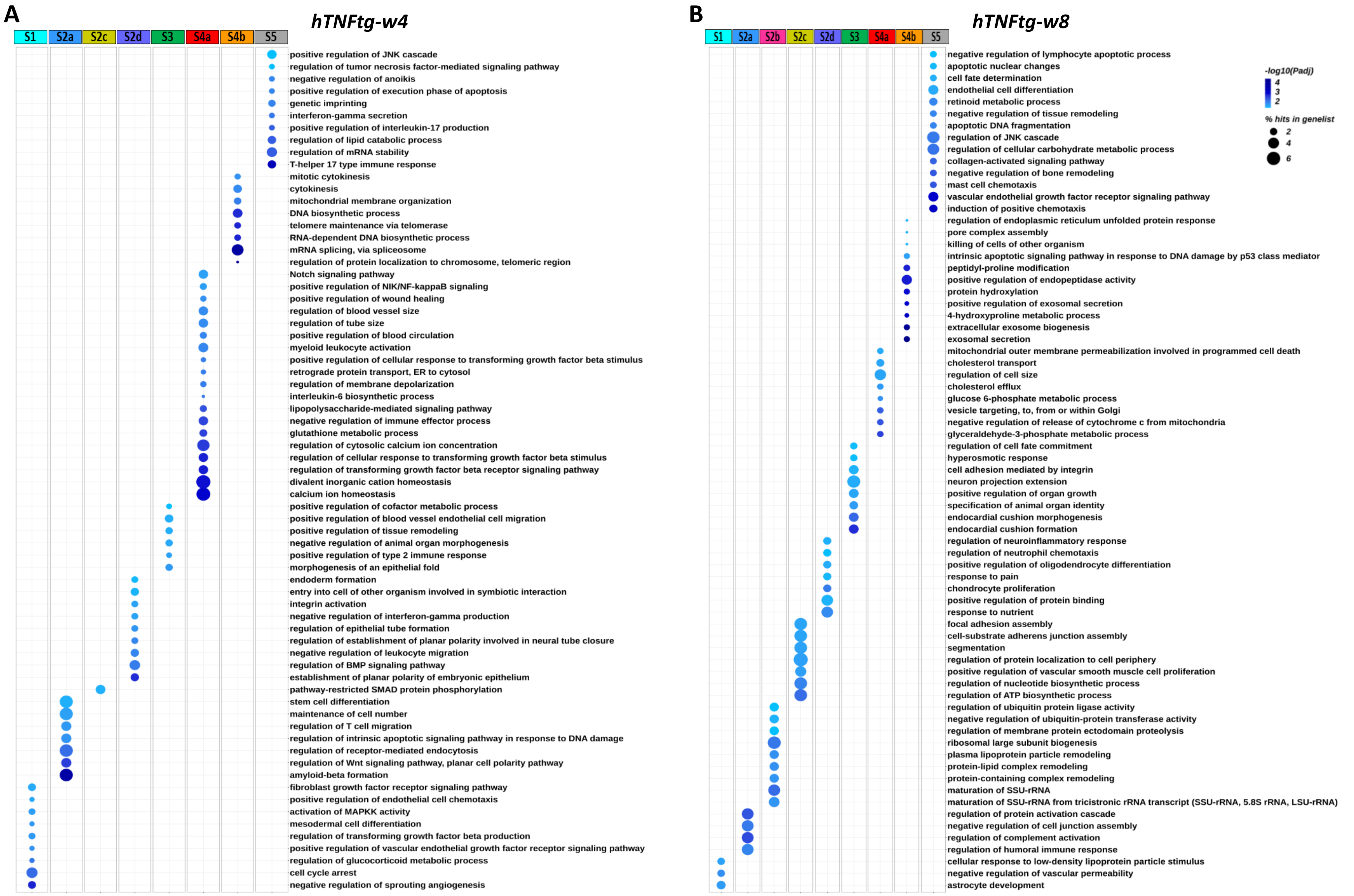
A
hTNFtg-w4
S1
S2a
S2c
S2d
S3
S4a
S4b
S5
positive regulation of JNK cascade
regulation of tumor necrosis factor-mediated signaling pathway
negative regulation of anoikis
positive regulation of execution phase of apoptosis
genetic imprinting
interferon-gamma secretion
positive regulation of interleukin-17 production
regulation of lipid catabolic process
regulation of mRNA stability
T-helper 17 type immune response
mitotic cytokinesis
cytokinesis
mitochondrial membrane organization
DNA biosynthetic process
telomere maintenance via telomerase
RNA-dependent DNA biosynthetic process
mRNA splicing, via spliceosome
regulation of protein localization to chromosome, telomeric region
Notch signaling pathway
positive regulation of NIK/NF-kappaB signaling
positive regulation of wound healing
regulation of blood vessel size
regulation of tube size
positive regulation of blood circulation
myeloid leukocyte activation
positive regulation of cellular response to transforming growth factor beta stimulus
retrograde protein transport, ER to cytosol
regulation of membrane depolarization
interleukin-6 biosynthetic process
lipopolysaccharide-mediated signaling pathway
negative regulation of immune effector process
glutathione metabolic process
regulation of cytosolic calcium ion concentration
regulation of cellular response to transforming growth factor beta stimulus
regulation of transforming growth factor beta receptor signaling pathway
divalent inorganic cation homeostasis
calcium ion homeostasis
positive regulation of cofactor metabolic process
positive regulation of blood vessel endothelial cell migration
positive regulation of tissue remodeling
negative regulation of animal organ morphogenesis
positive regulation of type 2 immune response
morphogenesis of an epithelial fold
endoderm formation
entry into cell of other organism involved in symbiotic interaction
integrin activation
negative regulation of interferon-gamma production
regulation of epithelial tube formation
regulation of establishment of planar polarity involved in neural tube closure
negative regulation of leukocyte migration
regulation of BMP signaling pathway
establishment of planar polarity of embryonic epithelium
pathway-restricted SMAD protein phosphorylation
stem cell differentiation
maintenance of cell number
regulation of T cell migration
regulation of intrinsic apoptotic signaling pathway in response to DNA damage
regulation of receptor-mediated endocytosis
regulation of Wnt signaling pathway, planar cell polarity pathway
amyloid-beta formation
fibroblast growth factor receptor signaling pathway
positive regulation of endothelial cell chemotaxis
activation of MAPKK activity
mesodermal cell differentiation
regulation of transforming growth factor beta production
positive regulation of vascular endothelial growth factor receptor signaling pathway
regulation of glucocorticoid metabolic process
cell cycle arrest
negative regulation of sprouting angiogenesis
B
hTNFtg-w8
S1
S2a
S2b
S2c
S2d
S3
S4a
S4b
S5
negative regulation of lymphocyte apoptotic process
apoptotic nuclear changes
cell fate determination
endothelial cell differentiation
retinoid metabolic process
negative regulation of tissue remodeling
apoptotic DNA fragmentation
regulation of JNK cascade
regulation of cellular carbohydrate metabolic process
collagen-activated signaling pathway
negative regulation of bone remodeling
mast cell chemotaxis
vascular endothelial growth factor receptor signaling pathway
induction of positive chemotaxis
regulation of endoplasmic reticulum unfolded protein response
pore complex assembly
killing of cells of other organism
intrinsic apoptotic signaling pathway in response to DNA damage by p53 class mediator
peptidyl-proline modification
positive regulation of endopeptidase activity
protein hydroxylation
positive regulation of exosomal secretion
4-hydroxyproline metabolic process
extracellular exosome biogenesis
exosomal secretion
mitochondrial outer membrane permeabilization involved in programmed cell death
cholesterol transport
regulation of cell size
cholesterol efflux
glucose 6-phosphate metabolic process
vesicle targeting, to, from or within Golgi
negative regulation of release of cytochrome c from mitochondria
glyceraldehyde-3-phosphate metabolic process
regulation of cell fate commitment
hyperosmotic response
cell adhesion mediated by integrin
neuron projection extension
positive regulation of organ growth
specification of animal organ identity
endocardial cushion morphogenesis
endocardial cushion formation
regulation of neuroinflammatory response
regulation of neutrophil chemotaxis
positive regulation of oligodendrocyte differentiation
response to pain
chondrocyte proliferation
positive regulation of protein binding
response to nutrient
focal adhesion assembly
cell-substrate adherens junction assembly
segmentation
regulation of protein localization to cell periphery
positive regulation of vascular smooth muscle cell proliferation
regulation of nucleotide biosynthetic process
regulation of ATP biosynthetic process
regulation of ubiquitin protein ligase activity
negative regulation of ubiquitin-protein transferase activity
regulation of membrane protein ectodomain proteolysis
ribosomal large subunit biogenesis
plasma lipoprotein particle remodeling
protein-lipid complex remodeling
protein-containing complex remodeling
maturation of SSU-rRNA
maturation of SSU-rRNA from tricistronic rRNA transcript (SSU-rRNA, 5.8S rRNA, LSU-rRNA)
regulation of protein activation cascade
negative regulation of cell junction assembly
regulation of complement activation
regulation of humoral immune response
cellular response to low-density lipoprotein particle stimulus
negative regulation of vascular permeability
astrocyte development
-log10(Padj)
4
3
2
% hits in genelist
2
4
6

Fig. S5

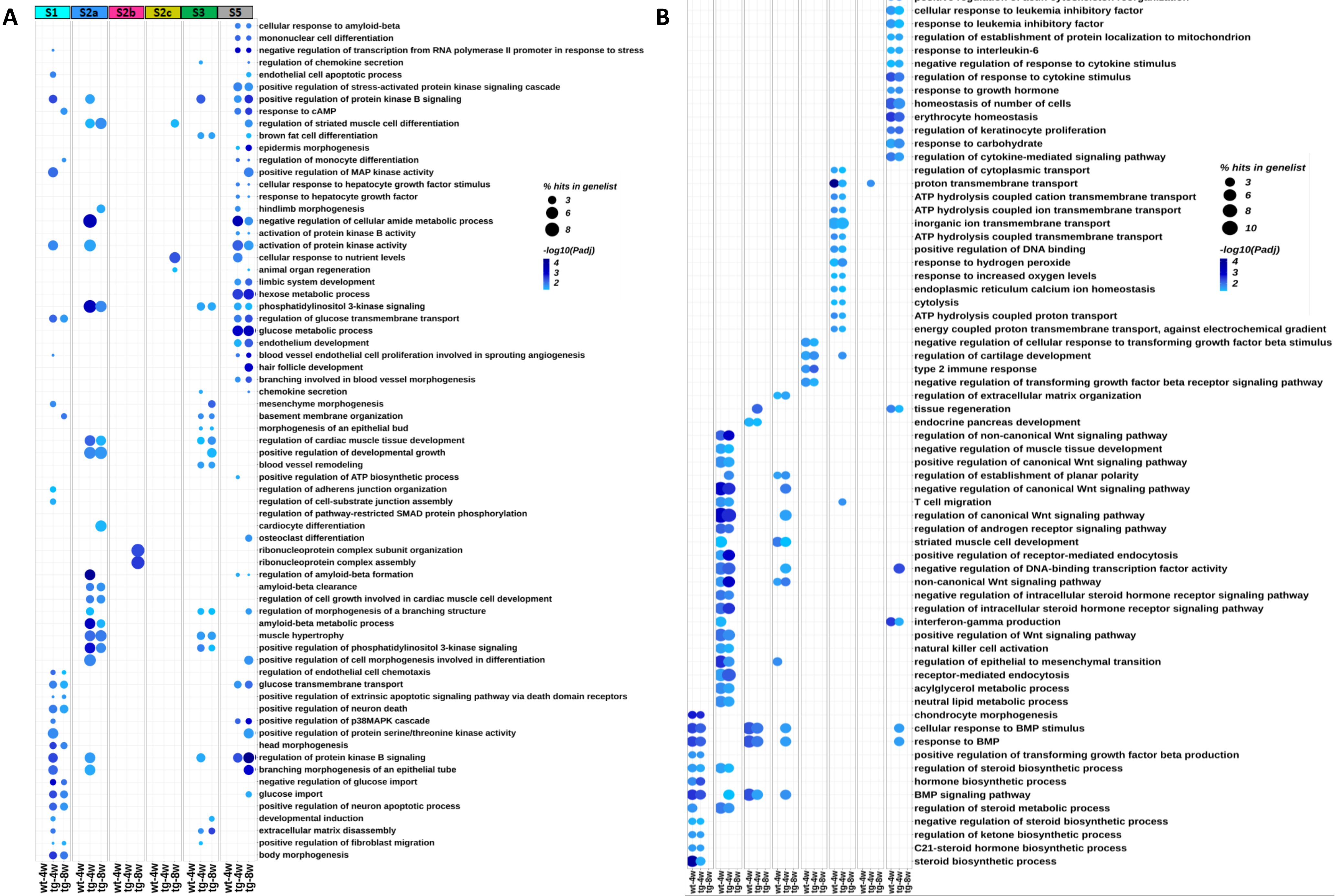
A
S1
S2a
S2b
S2c
S3
S5
cellular response to amyloid-beta
mononuclear cell differentiation
negative regulation of transcription from RNA polymerase II promoter in response to stress
regulation of chemokine secretion
endothelial cell apoptotic process
positive regulation of stress-activated protein kinase signaling cascade
positive regulation of protein kinase B signaling
response to cAMP
regulation of striated muscle cell differentiation
brown fat cell differentiation
epidermis morphogenesis
regulation of monocyte differentiation
positive regulation of MAP kinase activity
cellular response to hepatocyte growth factor stimulus
response to hepatocyte growth factor
hindlimb morphogenesis
negative regulation of cellular amide metabolic process
activation of protein kinase B activity
activation of protein kinase activity
cellular response to nutrient levels
animal organ regeneration
limbic system development
hexose metabolic process
phosphatidylinositol 3-kinase signaling
regulation of glucose transmembrane transport
glucose metabolic process
endothelium development
blood vessel endothelial cell proliferation involved in sprouting angiogenesis
hair follicle development
branching involved in blood vessel morphogenesis
chemokine secretion
mesenchyme morphogenesis
basement membrane organization
morphogenesis of an epithelial bud
regulation of cardiac muscle tissue development
positive regulation of developmental growth
blood vessel remodeling
positive regulation of ATP biosynthetic process
regulation of adherens junction organization
regulation of cell-substrate junction assembly
regulation of pathway-restricted SMAD protein phosphorylation
cardiocyte differentiation
osteoclast differentiation
ribonucleoprotein complex subunit organization
ribonucleoprotein complex assembly
regulation of amyloid-beta formation
amyloid-beta clearance
regulation of cell growth involved in cardiac muscle cell development
regulation of morphogenesis of a branching structure
amyloid-beta metabolic process
muscle hypertrophy
positive regulation of phosphatidylinositol 3-kinase signaling
positive regulation of cell morphogenesis involved in differentiation
regulation of endothelial cell chemotaxis
glucose transmembrane transport
positive regulation of extrinsic apoptotic signaling pathway via death domain receptors
positive regulation of neuron death
positive regulation of p38MAPK cascade
positive regulation of protein serine/threonine kinase activity
head morphogenesis
regulation of protein kinase B signaling
branching morphogenesis of an epithelial tube
negative regulation of glucose import
glucose import
positive regulation of neuron apoptotic process
developmental induction
extracellular matrix disassembly
positive regulation of fibroblast migration
body morphogenesis
% hits in genelist
3
6
8
-log10(Padj)
4
3
2
wt-4w tg-4w tg-8w
B
S1
S2a
S2c
S2d
S3
S4a
S4b
S5
positive regulation of actin cytoskeleton reorganization
cellular response to leukemia inhibitory factor
response to leukemia inhibitory factor
regulation of establishment of protein localization to mitochondrion
response to interleukin-6
negative regulation of response to cytokine stimulus
regulation of response to cytokine stimulus
response to growth hormone
homeostasis of number of cells
erythrocyte homeostasis
regulation of keratinocyte proliferation
response to carbohydrate
regulation of cytokine-mediated signaling pathway
regulation of cytoplasmic transport
proton transmembrane transport
ATP hydrolysis coupled cation transmembrane transport
ATP hydrolysis coupled ion transmembrane transport
inorganic ion transmembrane transport
ATP hydrolysis coupled transmembrane transport
positive regulation of DNA binding
response to hydrogen peroxide
response to increased oxygen levels
endoplasmic reticulum calcium ion homeostasis
cytolysis
ATP hydrolysis coupled proton transport
energy coupled proton transmembrane transport, against electrochemical gradient
negative regulation of cellular response to transforming growth factor beta stimulus
regulation of cartilage development
type 2 immune response
negative regulation of transforming growth factor beta receptor signaling pathway
regulation of extracellular matrix organization
tissue regeneration
endocrine pancreas development
regulation of non-canonical Wnt signaling pathway
negative regulation of muscle tissue development
positive regulation of canonical Wnt signaling pathway
regulation of establishment of planar polarity
negative regulation of canonical Wnt signaling pathway
T cell migration
regulation of canonical Wnt signaling pathway
regulation of androgen receptor signaling pathway
striated muscle cell development
positive regulation of receptor-mediated endocytosis
negative regulation of DNA-binding transcription factor activity
non-canonical Wnt signaling pathway
negative regulation of intracellular steroid hormone receptor signaling pathway
regulation of intracellular steroid hormone receptor signaling pathway
interferon-gamma production
positive regulation of Wnt signaling pathway
natural killer cell activation
regulation of epithelial to mesenchymal transition
receptor-mediated endocytosis
acylglycerol metabolic process
neutral lipid metabolic process
chondrocyte morphogenesis
cellular response to BMP stimulus
response to BMP
positive regulation of transforming growth factor beta production
regulation of steroid biosynthetic process
hormone biosynthetic process
BMP signaling pathway
regulation of steroid metabolic process
negative regulation of steroid biosynthetic process
regulation of ketone biosynthetic process
C21-steroid hormone biosynthetic process
steroid biosynthetic process
% hits in genelist
3
6
8
10
-log10(Padj)
4
3
2
wt-4w tg-4w tg-8w

Fig. S6

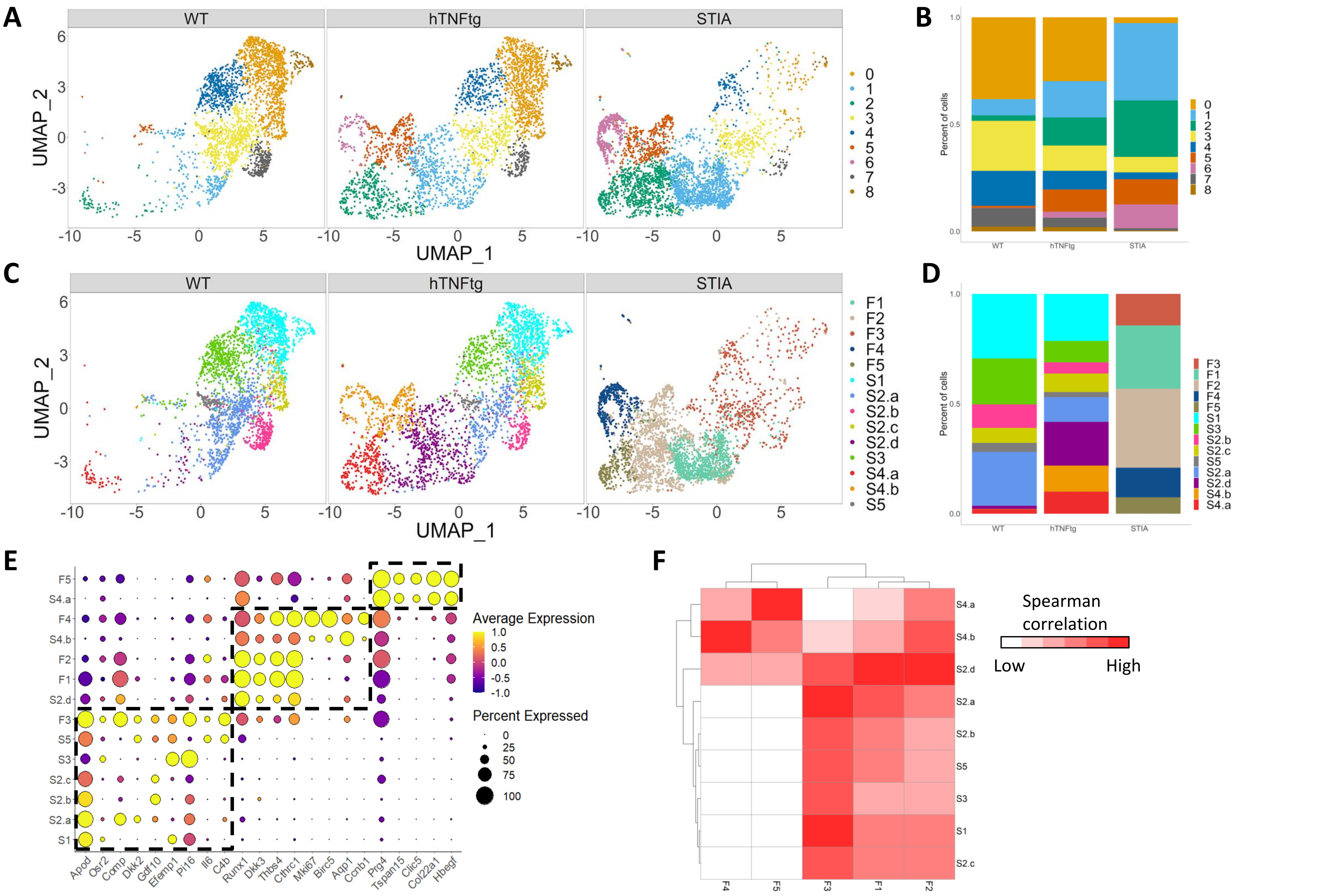
A
WT
hTNFtg
STIA
UMAP_2
UMAP_1
0
1
2
3
4
5
6
7
8
B
Percent of cells
C
F1
F2
F3
F4
F5
S1
S2.a
S2.b
S2.c
S2.d
S3
S4.a
S4.b
S5
D
E
Average Expression
Percent Expressed
Apod
Osr2
Comp
Dkk2
Gdf10
Efemp1
Pi16
Il6
C4b
Runx1
Dkk3
Thbs4
Cthrc1
Mki67
Birc5
Aqp1
Ccnb1
Prg4
Tspan15
Clic5
Col22a1
Hbegf
F
Spearman correlation
Low
High

Fig. S7

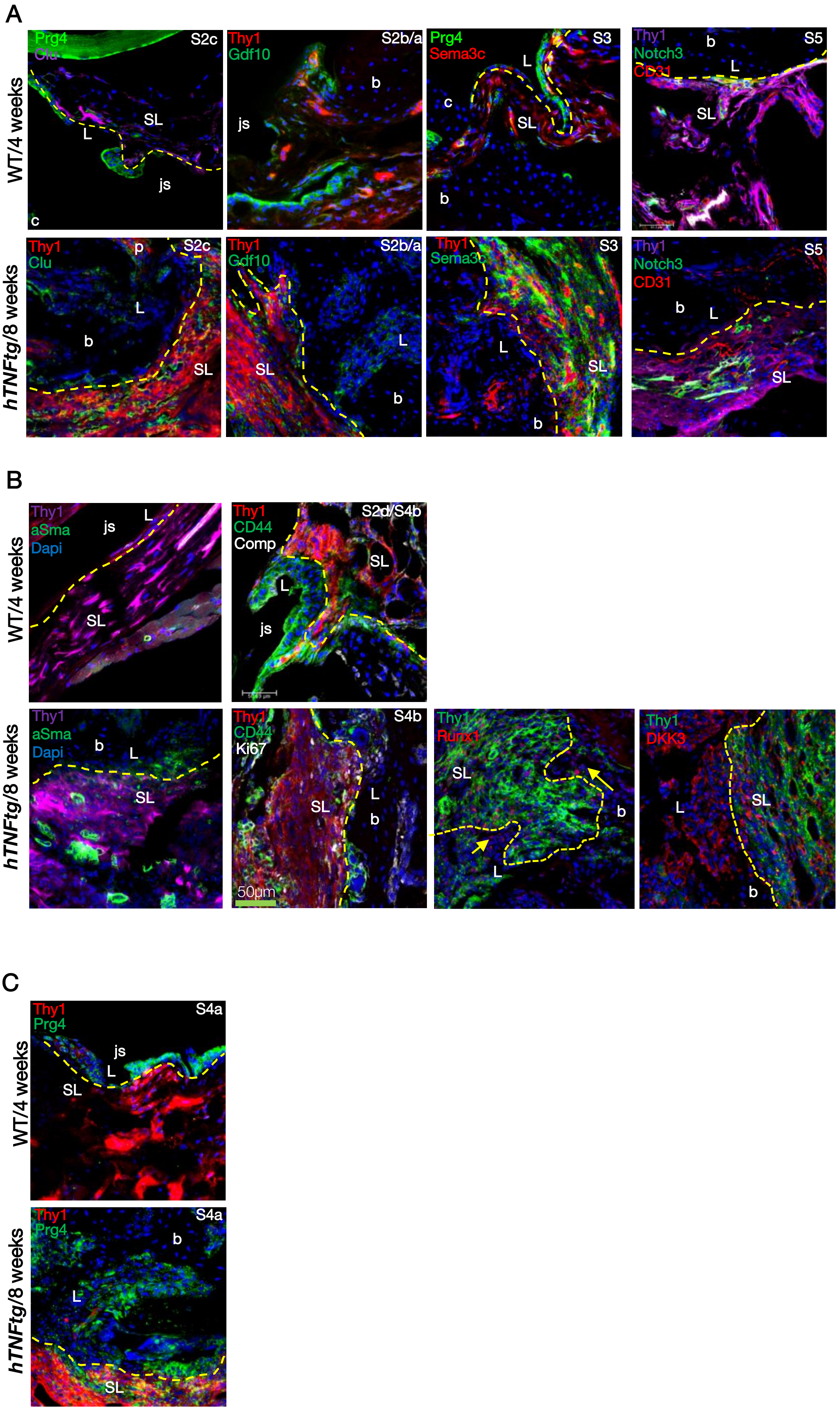
A
WT/4 weeks
hTNFtg/8 weeks
Prg4
Clu
S2c
SL
L
js
c
Thy1
Gdf10
S2b/a
b
Sema3c
S3
Notch3
CD31
S5
p
B
aSma
Dapi
CD44
Comp
S2d/S4b
Ki67
S4b
50µm
Runx1
DKK3
C
S4a

Figure S8

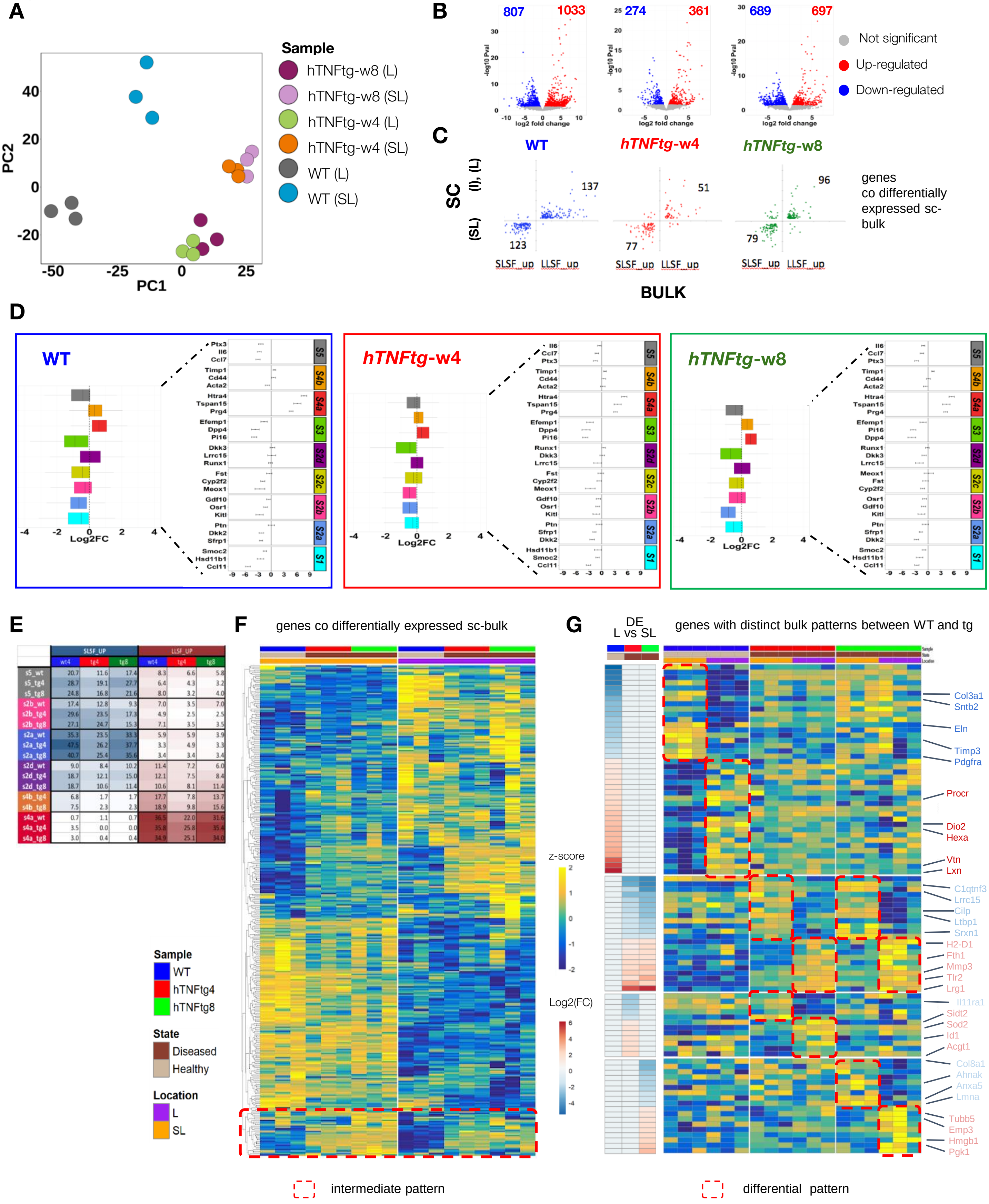

	SLSF_UP			LLSF_UP		
	wt4	tg4	tg8	wt4	tg4	tg8
s5_wt	20.7	11.6	17.4	8.3	6.6	5.8
s5_tg4	28.7	19.1	27.7	6.4	4.3	3.2
s5_tg8	24.8	16.8	21.6	8.0	3.2	4.0
s2b_wt	17.4	12.8	9.3	7.0	3.5	7.0
s2b_tg4	29.6	23.5	17.3	4.9	2.5	2.5
s2b_tg8	27.1	24.7	15.3	7.1	3.5	3.5
s2a_wt	35.3	23.5	33.3	5.9	5.9	3.9
s2a_tg4	47.5	26.2	37.7	3.3	4.9	3.3
s2a_tg8	40.7	25.4	35.6	3.4	3.4	3.4
s2d_wt	9.0	8.4	10.2	11.4	7.2	6.0
s2d_tg4	18.7	12.1	15.0	12.1	7.5	8.4
s2d_tg8	18.7	10.6	11.4	10.6	8.1	11.4
s4b_tg4	6.8	1.7	1.7	17.7	7.8	13.7
s4b_tg8	7.5	2.3	2.3	18.9	9.8	15.6
s4a_wt	0.7	1.1	0.7	36.5	22.0	31.6
s4a_tg4	3.5	0.0	0.0	35.8	25.8	35.4
s4a_tg8	3.0	0.4	0.4	34.9	25.1	34.0

Fig. S9

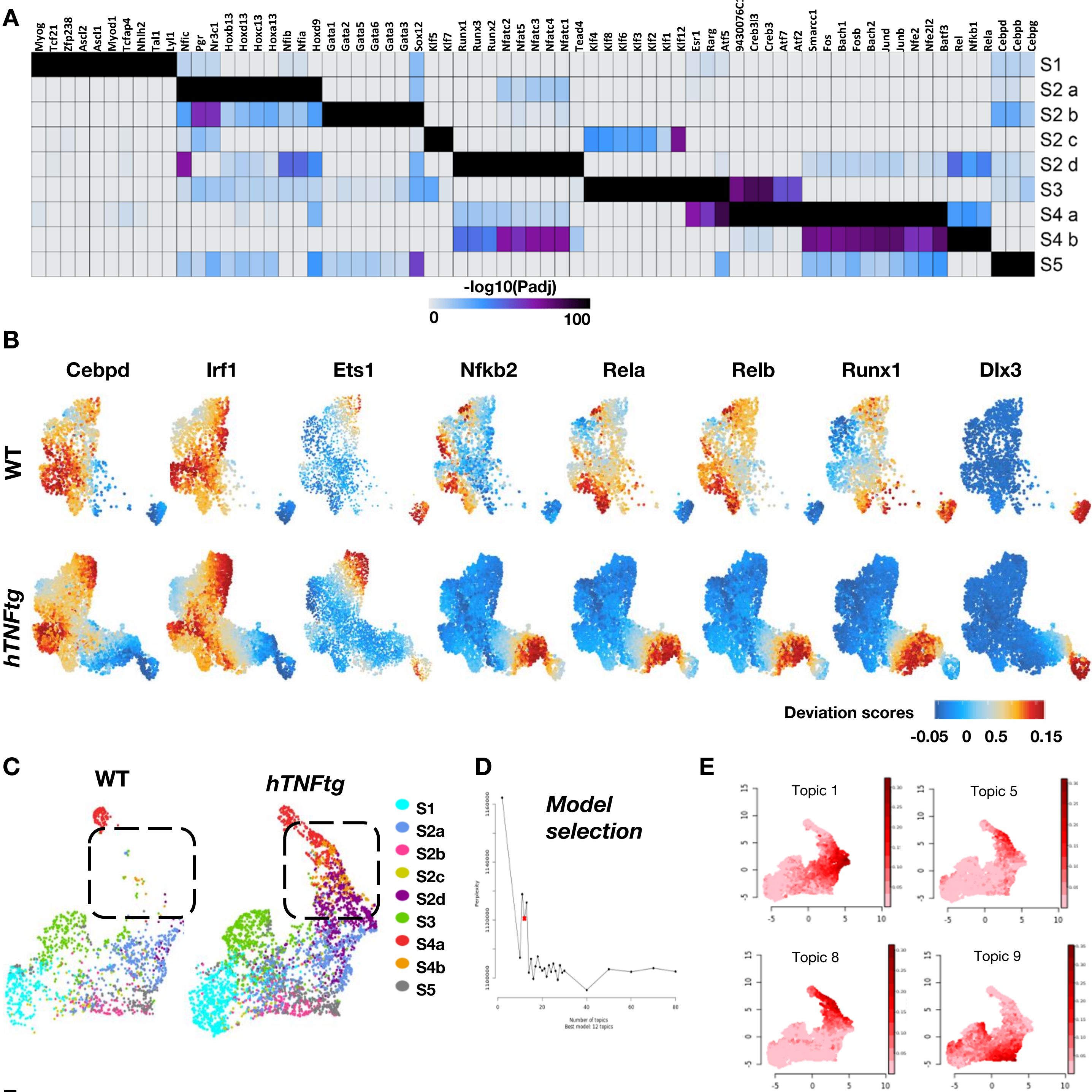

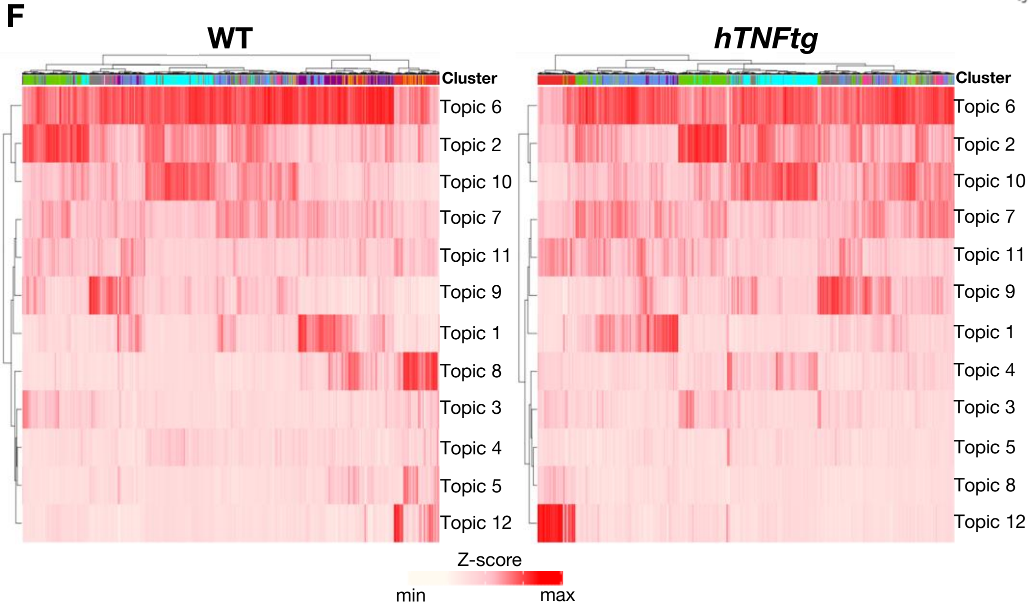

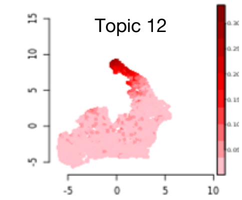

G

Topic	Transcription Factors
5	Bach1, Rel, Runx1, Nfe2l2, Cebpb
8	Atf3, Bach1,Runx1, Runx2, Klf1
12	Creb5, Atf7, c-Jun, Sp2, Dlx3

Fig. S10

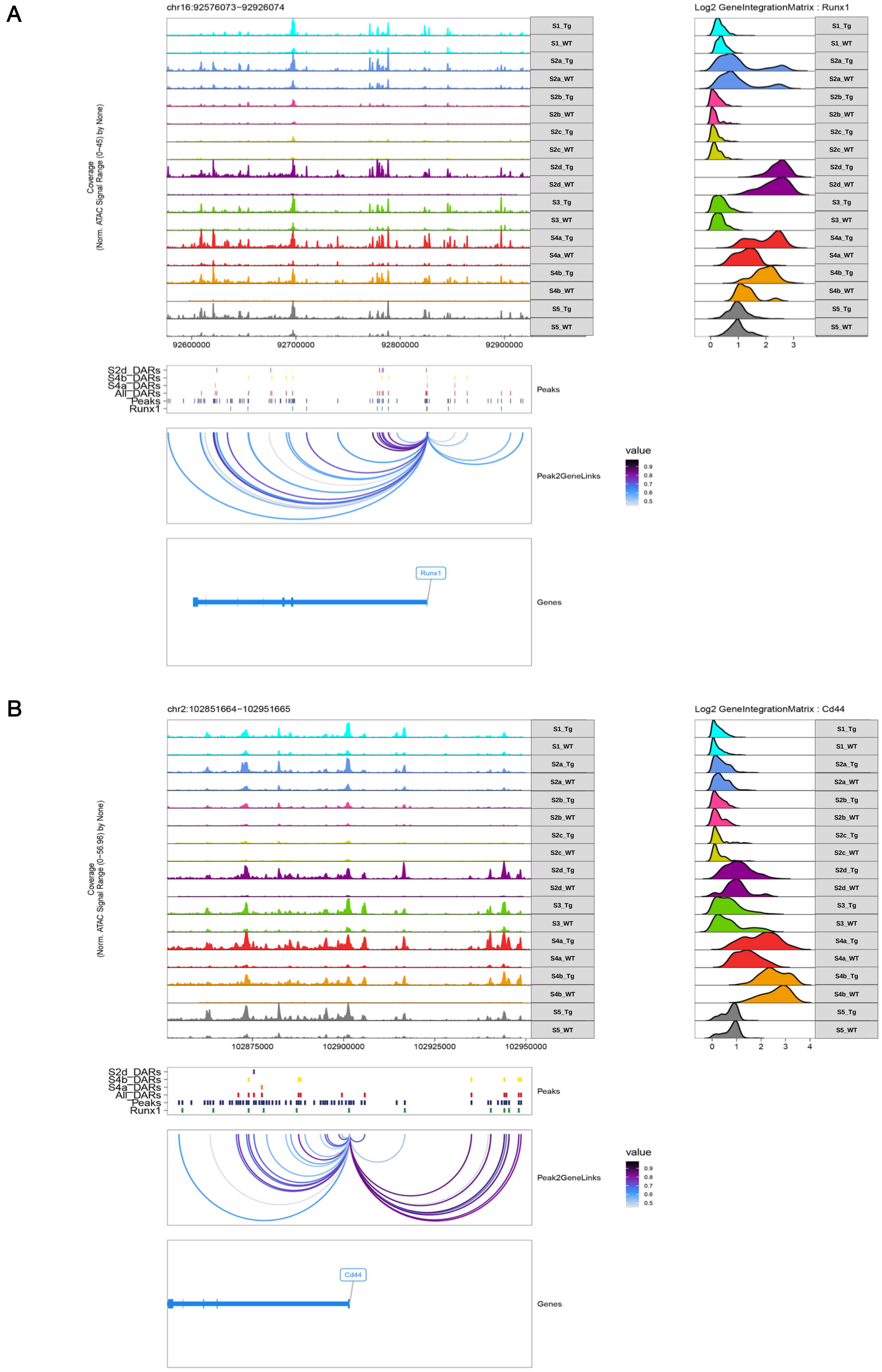
A
chr16:92576073−92926074
Coverage
(Norm. ATAC Signal Range (0−45) by None)
S1_Tg
S1_WT
S2a_Tg
S2a_WT
S2b_Tg
S2b_WT
S2c_Tg
S2c_WT
S2d_Tg
S2d_WT
S3_Tg
S3_WT
S4a_Tg
S4a_WT
S4b_Tg
S4b_WT
S5_Tg
S5_WT
92600000
92700000
92800000
92900000
Log2 GeneIntegrationMatrix : Runx1
0
1
2
3
S2d_DARs
S4b_DARs
S4a_DARs
All_DARs
Peaks
Runx1
Peaks
Peak2GeneLinks
value
0.9
0.8
0.7
0.6
0.5
Runx1
Genes
B
chr2:102851664−102951665
Coverage
(Norm. ATAC Signal Range (0−56.96) by None)
102875000
102900000
102925000
102950000
Log2 GeneIntegrationMatrix : Cd44
0
1
2
3
4
Cd44

Fig. S11

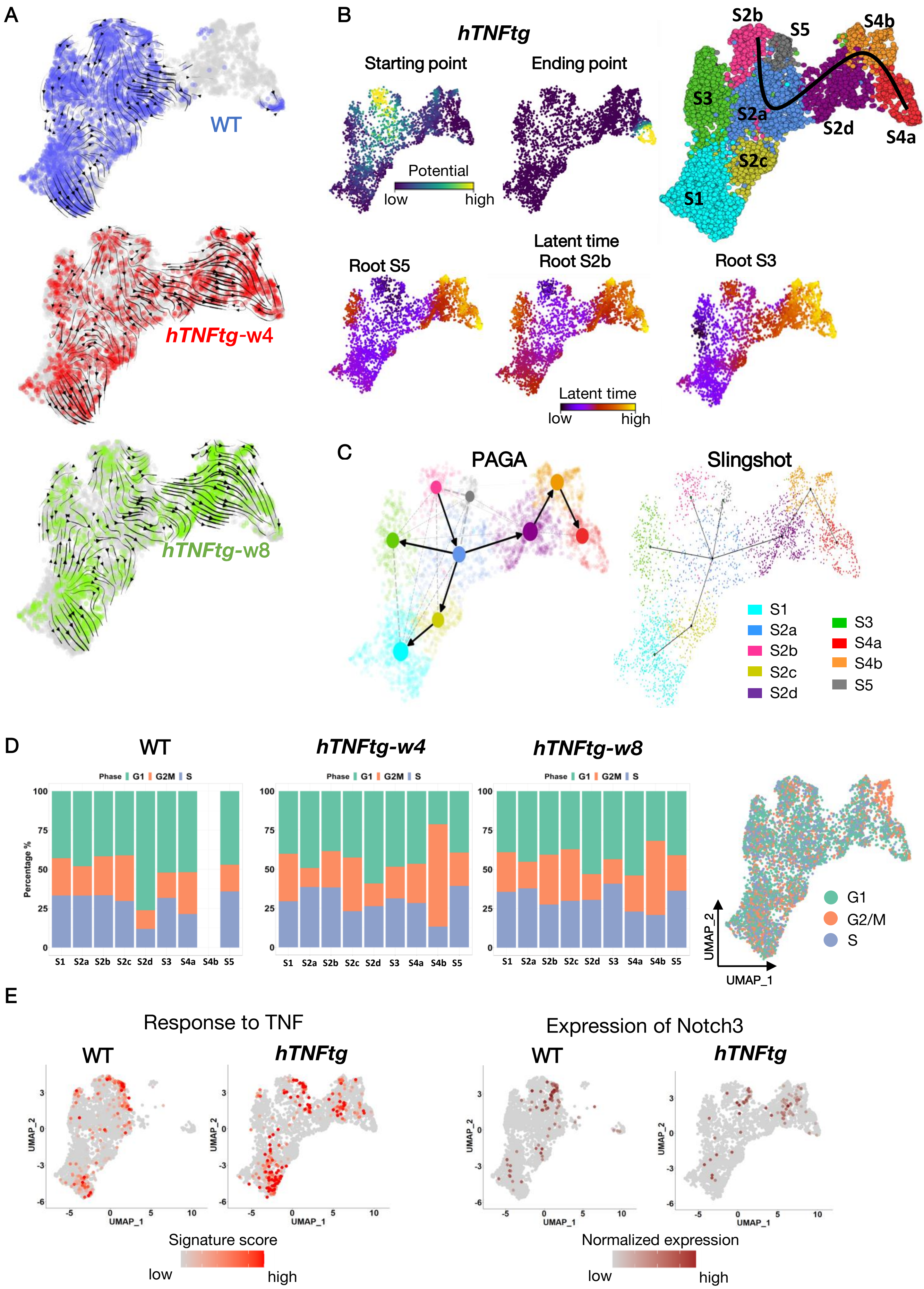
A
WT
hTNFtg-w4
hTNFtg-w8
B
hTNFtg
Starting point
Ending point
Potential
low
high
S2b
S5
S4b
S3
S2a
S2d
S4a
S2c
S1
Root S5
Latent time
Root S2b
Root S3
Latent time
low
high
C
PAGA
Slingshot
S1
S2a
S2b
S2c
S2d
S3
S4a
S4b
S5
D
WT
hTNFtg-w4
hTNFtg-w8
Phase G1 G2M S
Percentage %
G1
G2/M
S
UMAP_2
UMAP_1
E
Response to TNF
WT
hTNFtg
Expression of Notch3
WT
hTNFtg
Signature score
low
high
Normalized expression
low
high

Fig. S12

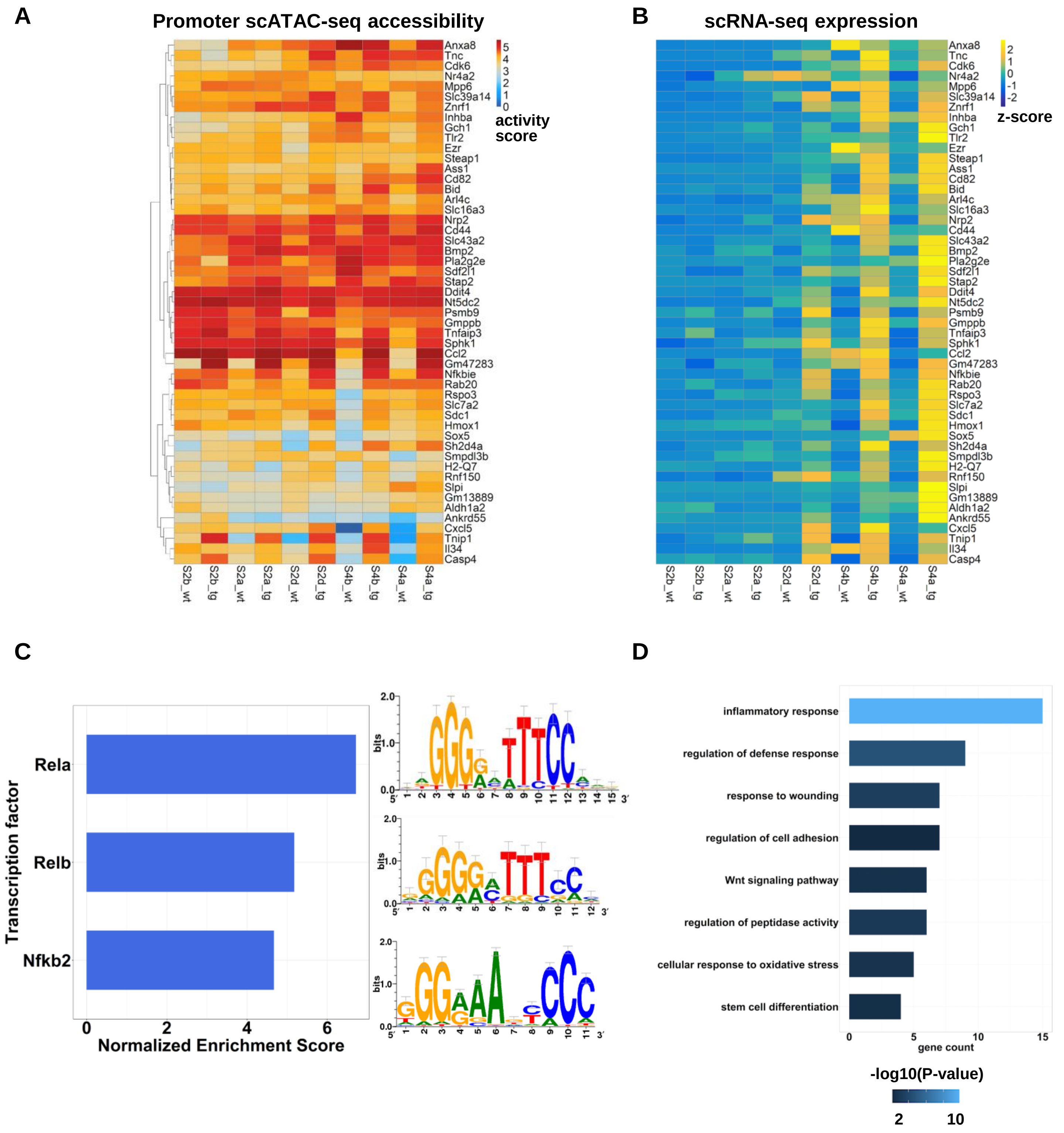
A
Promoter scATAC-seq accessibility
activity score
B
scRNA-seq expression
z-score
Anxa8
Tnc
Cdk6
Nr4a2
Mpp6
Slc39a14
Znrf1
Inhba
Gch1
Tlr2
Ezr
Steap1
Ass1
Cd82
Bid
Arl4c
Slc16a3
Nrp2
Cd44
Slc43a2
Bmp2
Pla2g2e
Sdf2l1
Stap2
Ddit4
Nt5dc2
Psmb9
Gmppb
Tnfaip3
Sphk1
Ccl2
Gm47283
Nfkbie
Rab20
Rspo3
Slc7a2
Sdc1
Hmox1
Sox5
Sh2d4a
Smpdl3b
H2-Q7
Rnf150
Slpi
Gm13889
Aldh1a2
Ankrd55
Cxcl5
Tnip1
Il34
Casp4
S2b_wt
S2b_tg
S2a_wt
S2a_tg
S2d_wt
S2d_tg
S4b_wt
S4b_tg
S4a_wt
S4a_tg
C
Transcription factor
Rela
Relb
Nfkb2
Normalized Enrichment Score
bits
D
inflammatory response
regulation of defense response
response to wounding
regulation of cell adhesion
Wnt signaling pathway
regulation of peptidase activity
cellular response to oxidative stress
stem cell differentiation
gene count
-log10(P-value)

Fig. S13

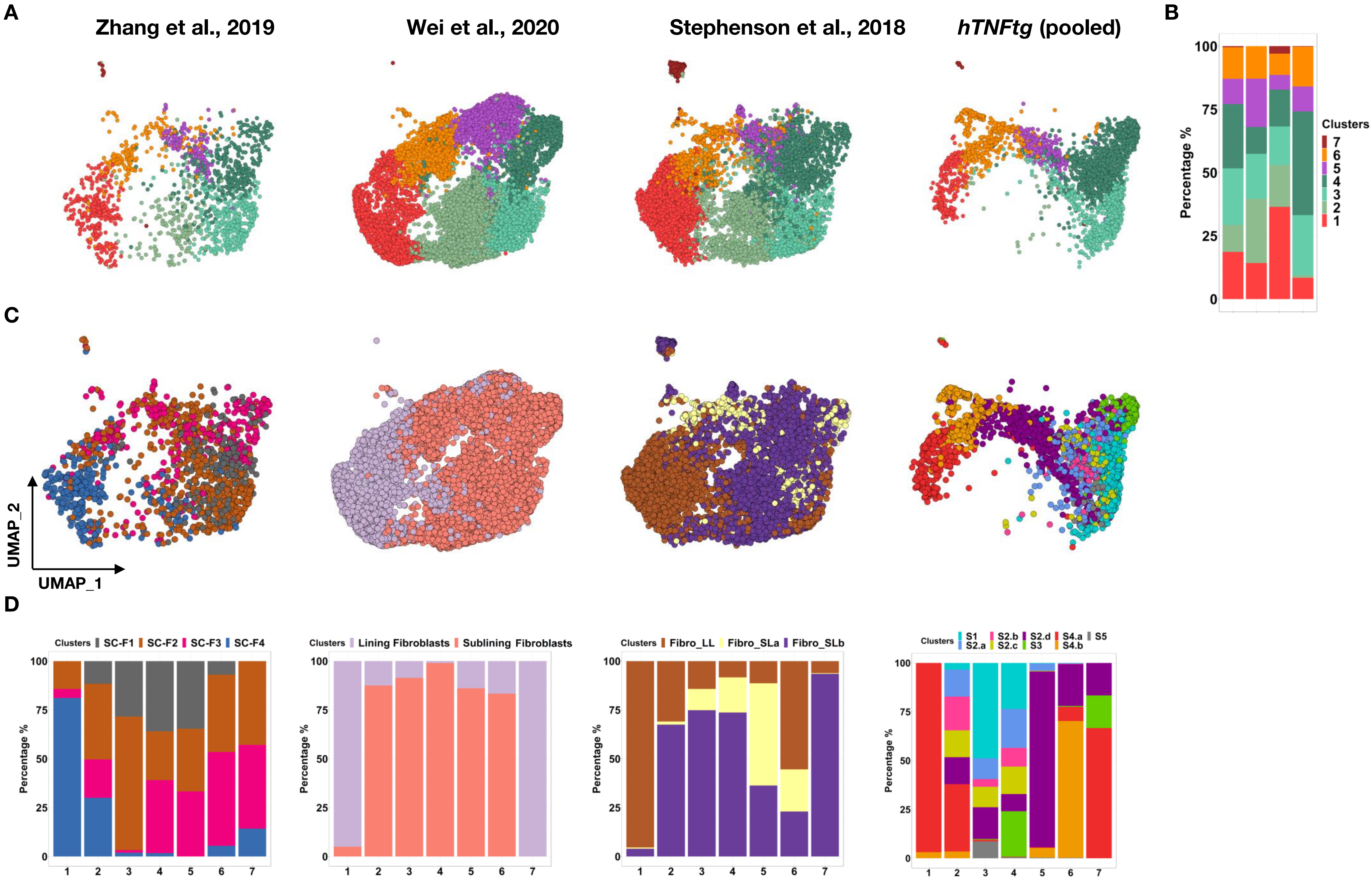
A
Zhang et al., 2019
Wei et al., 2020
Stephenson et al., 2018
hTNFtg (pooled)
B
Percentage %
Clusters
7
6
5
4
3
2
1
100
75
50
25
0
C
UMAP_2
UMAP_1
D
Clusters SC-F1 SC-F2 SC-F3 SC-F4
Clusters Lining Fibroblasts Sublining Fibroblasts
Clusters Fibro_LL Fibro_SLa Fibro_SLb
Clusters S1 S2.a S2.b S2.c S2.d S3 S4.a S4.b S5
Percentage %
1 2 3 4 5 6 7

Fig. S14

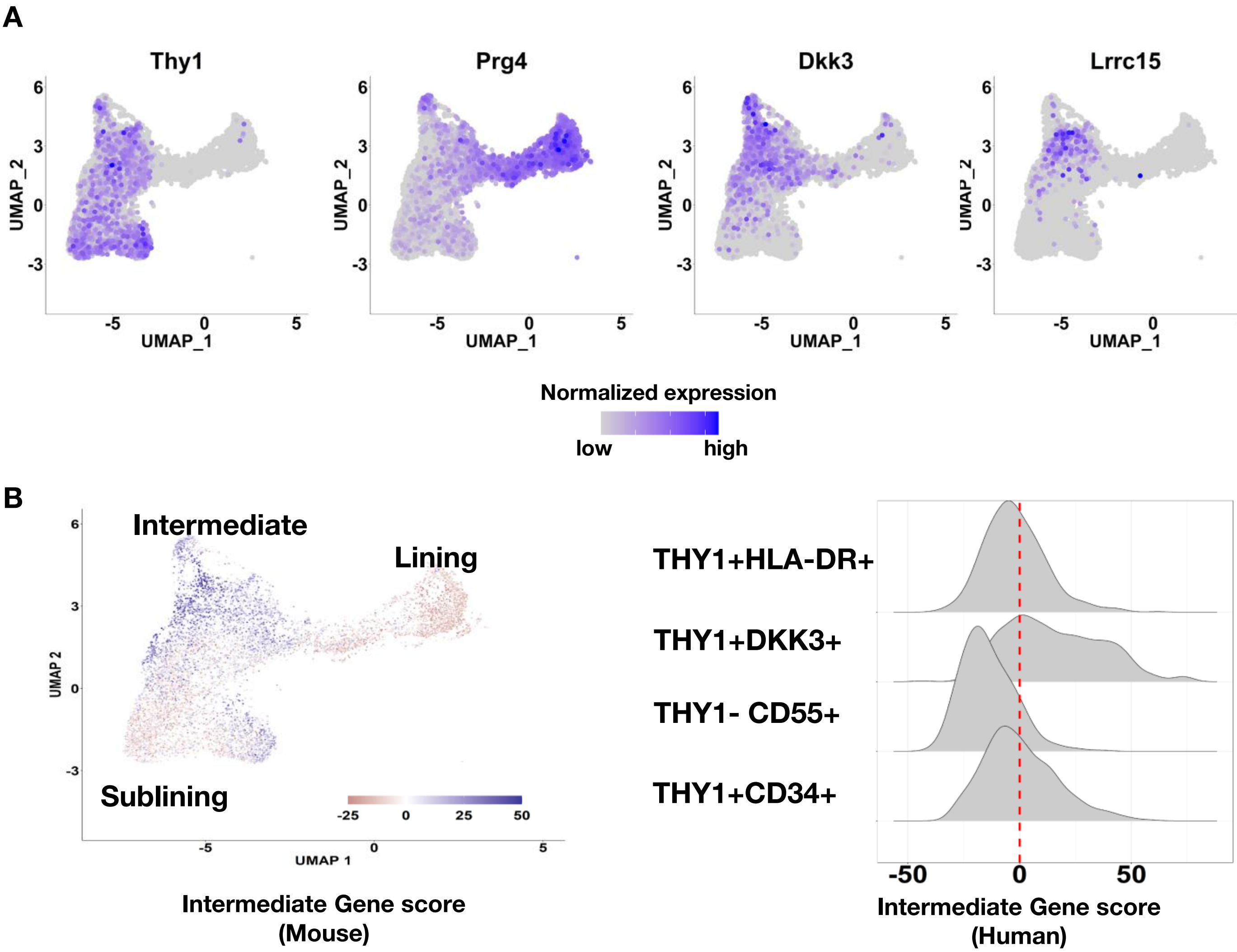

A
Thy1
Prg4
Dkk3
Lrrc15
UMAP_2
UMAP_1
Normalized expression
low
high
B
Intermediate
Lining
Sublining
UMAP 2
UMAP 1
Intermediate Gene score
(Mouse)
THY1+HLA-DR+
THY1+DKK3+
THY1- CD55+
THY1+CD34+
Intermediate Gene score
(Human)
